# Premature differentiation of nephron progenitor cell and dysregulation of gene pathways critical to kidney development in a model of preterm birth

**DOI:** 10.1038/s41598-021-00489-y

**Published:** 2021-11-04

**Authors:** Aleksandra Cwiek, Masako Suzuki, Kimberly deRonde, Mark Conaway, Kevin M. Bennett, Samir El Dahr, Kimberly J. Reidy, Jennifer R. Charlton

**Affiliations:** 1 Division of Nephrology, Department of Pediatrics, University of Virginia, Box 800386, Charlottesville, VA 22903 USA; 2 Division of Nephrology, Department of Pediatrics, Children’s Hospital at Montefiore, New York, NY USA; 3 Department of Genetics, Albert Einstein College of Medicine, New York, NY USA; 4 University of Virginia Health System, Charlottesville, VA USA; 5 Division of Translational Research and Applied Statistics, Department of Public Health Sciences, University of Virginia School of Medicine, University of Virginia, Charlottesville, VA USA; 6 Mallinckrodt Institute of Radiology, Washington University School of Medicine, St. Louis, MO USA; 7 Department of Pediatrics, Tulane University School of Medicine and Children’s Hospital of New Orleans, New Orleans, LA USA; 8 Cell & Developmental Biology Graduate Program, University of Virginia School of Medicine, Charlottesville, VA 22903 USA

**Keywords:** Medical research, Preclinical research

## Abstract

Preterm birth is a leading cause of neonatal morbidity. Survivors have a greater risk for kidney dysfunction and hypertension. Little is known about the molecular changes that occur in the kidney of individuals born preterm. Here, we demonstrate that mice delivered two days prior to full term gestation undergo premature cessation of nephrogenesis, resulting in a lower glomerular density. Kidneys from preterm and term groups exhibited differences in gene expression profiles at 20- and 27-days post-conception, including significant differences in the expression of fat-soluble vitamin-related genes. Kidneys of the preterm mice exhibited decreased proportions of endothelial cells and a lower expression of genes promoting angiogenesis compared to the term group. Kidneys from the preterm mice also had altered nephron progenitor subpopulations, early Six2 depletion, and altered Jag1 expression in the nephrogenic zone, consistent with premature differentiation of nephron progenitor cells. In conclusion, preterm birth alone was sufficient to shorten the duration of nephrogenesis and cause premature differentiation of nephron progenitor cells. These candidate genes and pathways may provide targets to improve kidney health in preterm infants.

## Introduction

Significant advances have been made in neonatal medicine over the past three decades. Neonates can be considered viable in the 22nd week of gestation^1,2^. These preterm neonates form nearly half of their lifetime supply of nephrons in an ex utero environment^3^. Nephrogenesis occurs through a complex interaction between the branching ureteric bud and the metanephric mesenchyme. In humans, a self-renewing population of nephron progenitor cells in the metanephric mesenchyme niche forms new nephrons until 32–36 weeks^3–5^. These progenitor cells self-renew and differentiate, forming structures from the glomerulus to the collecting duct. However, common ex utero exposures to nephrotoxic medications, hypoxia, and hyperoxia, are unfavorable for nephrogenesis^6,7^ and are associated with poor short-term outcomes in the kidney, such as acute kidney injury^8,9^.

Preterm neonates who are discharged from the neonatal intensive care unit also have a greater risk for poor long term kidney outcomes such as chronic kidney disease (CKD)^10–13^. Adults born preterm have an increased risk for developing CKD, detectable as early as childhood^10–12,14^. Infants born preterm are also at increased risk of high blood pressure and hypertension during childhood^14–20^. Although growing evidence suggests a significant connection between preterm birth and CKD, it is challenging to study the direct effect of preterm birth on long-term kidney outcomes. A great deal is unknown due to highly variable monitoring practices and a lack of evidence-based guidelines for monitoring kidney health after discharge from the neonatal intensive care unit.

Early histologic changes provide clues to the pathogenesis of kidney disease in primates and humans born preterm^21,22^. The nephrogenic zone, the niche for the progenitor cell population and immature glomerular structures, is significantly smaller in the preterm baboons^22^. Similarly, the nephrogenic zone is present for a shorter duration in the preterm humans^4^. Abnormal glomeruli are observed in the superficial cortex in both of these groups. Many of the glomeruli exhibit a cystic Bowman’s space and a shrunken tuft. In a case series of adolescents and adults born preterm who underwent a kidney biopsy for clinical indications, the major histologic finding was secondary focal segmental glomerulosclerosis^23^.

Several animal models of preterm birth have been developed, but research on the effect of preterm birth on the kidney is limited due to species-dependent kidney development and the resources necessary to conduct long-term studies in large animal models. Although baboons have a long gestational window with nephrogenesis complete prior to birth^24^, consistent with humans, the resources required to maintain a preterm baboon neonatal intensive care unit are prohibitive. Mouse, rat, and rabbit models have also been used to study preterm birth^25,26^, but the natural window of postnatal nephrogenesis is different in each species (mice: 4 days, rats: 7–10 days, rabbits: 3 weeks)^27^. In 2012, Stelloh et al.^18^ examined the effect of preterm birth on the kidneys of mice delivered two days before the end of gestation. By adulthood, the mice developed CKD exhibiting higher blood pressures, albuminuria, and lower glomerular filtration rates.

Here, we used the same preterm mouse model to examine the histologic and molecular changes in the kidney that occur shortly after birth to identify pathways that may be therapeutic targets to prevent CKD associated with preterm birth. We found that nephrogenesis in preterm mice terminated a full day earlier than in term mice. Preterm birth led to early differentiation of nephron progenitors and decreased podocyte and endothelial cell proportions. There was dysregulation of several gene pathways critical in kidney health, such as fat-soluble vitamins, angiogenesis, and immune cells.

## Methods

### Animals

All animal experiments were approved at the University of Virginia by the Institutional Animal Care and Use Committee in agreement with the National Institutes of Health Guide for the Care and Use of Laboratory Animals. The study is reported in accordance with ARRIVE guidelines. Timed pregnant CD-1 dams were purchased from Charles River Laboratory (Wilmington, MA). The Charles River timed mating protocol pairs a proven male breeder with a female overnight. The following morning is designated as day 1 of gestation if a vaginal plug is observed in the female mouse. The preterm group was comprised of pups delivered by Cesarean section at 18 days post-conception (18 dpc). This timepoint was chosen because previous investigators showed that delivery at this timepoint resulted in an adult phenotype with fewer glomeruli, lower glomerular filtration rate and albuminuria^18^. There were no published reports of pups surviving prior to this gestational age. We estimate that pups born at 18 dpc model a preterm human neonate at approximately 27 week gestation based on glomerular number. The dams were euthanized by cervical dislocation before the Cesarean section as previously published^18,28^. The abdomen was prepared with isopropyl alcohol prior to incision. Sterilized instruments were used. Following a midline incision, the uterine horns were removed and the offspring were carefully expelled from their amniotic sacs. The placenta was separated from each pup using forceps to compress the umbilical cord. The pups were dried and gently stimulated with cotton-tipped applicators. They were placed in an incubator at 37 °C and provided oxygen (95%) until their skin became pink. The average time from cervical dislocation to delivery of all pups was approximately 5 min but varied depending on the size of the liter. The preterm pups were placed with a foster mother after her own pups were removed and euthanized. Acepromazine maleate (AceproJect 10 mg/ml, Henry Schein Animal Health) with a final concentration of 75 mg/ml was added to the water of all mothers to reduce the risk of neglection or cannibalization for a total of 48 h in both groups. The average perinatal mortality rate of the pups was 20%. The term group of control animals was comprised of pups that were delivered vaginally at 20 ± 0.25 dpc. The pups were euthanized daily from 18 to 27 dpc in the preterm group (n = 3–11/group) and from 20 to 27 dpc in the term group (n = 3–16/group). The kidneys were collected and weighed, and the right kidney was stored in RNAlater™ Stabilization Solution (ThermoFisher Scientific, AM7020) for gene expression analyses.

### RNA extraction

RNA was extracted from the whole right kidney from the mice at 18 dpc (n = 3), 20 dpc (preterm n = 3, term n = 3) and 27 dpc (preterm n = 3, term n = 6) with TRIZOL (Invitrogen™, 15596018) and cleaned with RNeasy MinElute Cleanup Kit (Qiagen, 74204) with RNase-Free DNase Set (Qiagen, 79254). Eluted RNA was reconstituted with RNase-free water to a final volume of 50 µl and stored in − 80 °C until analysis. RNA concentration was quantified and quality was assessed with a NanoDrop™ One/OneC Microvolume UV–Vis Spectrophotometer (Thermo Fisher Scientific).

### RNA-sequencing

Gene expression profiles of the mouse kidneys from the preterm and term groups were evaluated by RNA-Seq on oligo-dT enriched mRNA using NEBNext^®^ Ultra™ RNA Library Prep Kits (non-directional) for Illumina, following manufacturer protocols. RNA sequencing was performed using the extracted RNA. The generated libraries were sequenced on NovaSeq600 with 150 bp paired-end sequencing. The quality of the sequences was assessed by FASTQC (http://www.bioinformatics.babraham.ac.uk/projects/fastqc/), and the library qualities were evaluated with RSeQC^29^ before the analysis. The obtained sequences were aligned to the mouse genome (mm10) with GenCode vM15 as a reference annotation using STAR aligner^30^. We counted the aligned reads per transcript for further analysis. The sequencing statistics are provided in Supplementary Table S1. We used DEseq2 to identify the differentially expressed genes of the animal, including sex genes^31^. Each analysis was adjusted for the sex of the offspring determined by the expression of Y chromosome genes. The list of Y chromosome genes was obtained using the UCSC Genome Browser Table Browser function. Genes with at least a two-fold difference with a false discovery rate (FDR) adjusted p < 0.05 were considered differentially expressed. The Gene Ontology enrichment analyses were implemented by the ClusterProfiler Bioconductor package at significance level q < 0.05^37^. The volcano plots were plotted − log10 FDR-adjusted p-values and log2-fold changes between groups using the Enhanced Volcano Bioconductor package (Supplementary Fig. S1). All statistical analyses were performed using R (version 4.0.2).

### Estimating cell subtype proportion of mouse kidneys from the RNA-seq data

The cell subtype proportions were estimated based on the gene expression profiles of 20 dpc preterm and term RNA-seq with a publicly available single-cell RNA-Seq (scRNA-seq) dataset of 20 dpc mouse kidney CD-1 mice (GSM2473317)^32^ using CIBERSORTx algorithm^33^. The scRNA-seq data were re-analyzed before the CIBERSORTx analysis with Seurat to identify clusters of cell types and the expression status of the clusters^34^. The cell subtypes of each cluster were identified by the expression status of the marker genes^32^. Identified clusters with the gene expression status of each cell were used to create a signature expression profile. The generated signature profile was used to estimate fractions of cell subtypes from each RNA-seq dataset. We used the S-mode batch correction with 100 permutations to generate the signature profile. The cell cycle of each cell was estimated by the CellCycleScoring function of Seurat using the expression status of cell-cycle specific genes, and pseudotime analysis was performed to assess the development time of the nephron progenitors proximal tubules and distal tubules by Monocle 3 algorithm^35^.

### Candidate gene analysis

We applied a candidate gene approach to analyze the RNA-seq data. The candidate gene list provided in Supplementary Table S2 was generated a priori from the literature. The genes of interest were categorized into three groups: (1) important in kidney development, (2) specific to a nephron segment, or (3) involved in kidney development pathways. We developed 30 categories within these three groups. Genes were categorized based on the location of gene expression and each gene was assigned to only one category. We ranked the categories by the number of genes differentially expressed between the preterm and term groups. The gene expression of the preterm group was compared to the term group at both 20 and 27 dpc. We conducted an Anderson–Darling (A–D) test for normality for each of the genes. The A–D test is designed to detect deviations from normality and thus generally has greater statistical power to detect non-normality than the Shapiro–Wilk statistic. These test results gave us no reason to doubt the normality assumptions. For 4.7% of the genes, the A–D test rejected normality at the 5% level of significance, almost exactly what would be expected if the normal assumption were valid. None of the A–D tests were significant using a false discovery rate of 5%. We corrected for multiple comparisons using a false discovery rate of 0.05. The p values were derived from the two-sample t-test with pooled variance. Point estimates and 95% confidence interval (95% CI) were calculated for each gene. Multivariate analysis without adjustments was conducted on each category of genes to compare point estimates from the preterm and term groups at both 20 and 27 dpc.

### Quantitative reverse transcription (RT)‐PCR

To verify the RNA-Seq findings, we assessed the gene expression status using reverse-transcription, followed by quantitative PCR. A total of 1 μg of RNA was reverse‐transcribed with MultiScribe™ Reverse Transcriptase (Thermo Fisher Scientific) to synthesize first‐strand cDNA. PCR mixtures (10 μl) containing cDNA template equivalent to 10 ng total RNA, 5 μM of gene-specific primer pairs, and PowerUp SYBR Green Master Mix (Thermo Fisher Scientific) were prepared and subject to PCR amplification using the CFX Connect Real-Time PCR Detection System (Bio‐Rad). The gene-specific primer pairs for *Acy3*, *Crabp1*, *Cyp24a1*, *Cyp27b1*, *Pfdn4*, *Wnt11*, *Slc34a1*, *Nog, Etv5,* and *S14* were designed with Primer 3 (http://bioinfo.ut.ee/primer3-0.4.0). The primer sequences and conditions of PCR amplification are listed in Supplementary Table S3. S14, (a housekeeping gene commonly used in studies of kidney development^36^), was used as a reference gene to assess the relative changes in gene expression. Data are reported as medians and interquartile ranges and analyzed using a t-test with a p-value < 0.05 being considered statistically significant.

### Histology and immunohistochemistry

The left kidney was bisected along the transverse plane and immersion fixed in 10% buffered formalin for 24 h, embedded in paraffin, and sectioned at 4 μm. Kidney sections were deparaffinized with xylene, followed by a rehydration procedure in a descending series of ethanol concentrations. Kidney sections stained with Periodic acid Schiff (PAS) or *Lotus tetragonolobus* lectin (LTL) were imaged using Grundium Ocus with a 20 × objective (© Grundium Ltd 2019). For histologic and immunohistochemical assessments, males and females were analyzed together because gene expression changes did not differ by sex in the day 20 RNA Seq data set. Further, Stelloh^18^ et al. did not find differences by sex in their analyses.

#### Nephrogenic zone (18–26 dpc)

Biotinylated LTL (Vector Laboratories, Burlingame, CA, USA, B1325, 1:50 dilution) was used to identify mature proximal tubules to determine cessation of the nephrogenic zone and identify immature structures (vesicles along with S-shaped and comma-shaped structures). To perform LTL staining, endogenous peroxidase was quenched by submersion of the slides in 30% hydrogen peroxide in methanol for 30 min. The kidney sections were treated for 30 min by enzymatic digestion by proteinase K in 0.1 M Na-PO_4_ buffer, then with biotinylated LTL for 1 h. Following incubation, the sections were washed twice for 5 min in phosphate buffer and incubated in Vectastain ABC reagent for 30 min. The sections were then incubated with 3,3′-diaminobenzidine (DAB), counterstained with a 0.5% methylene blue solution, and dehydrated. The nephrogenic zone was assessed in LTL-stained kidneys in both the preterm and term groups from 18 to 26 dpc. The nephrogenic zone was considered depleted when the mesenchymal cap cells were absent and the LTL positive cells of the proximal tubules reached the capsule. The presence of immature glomerular structures (vesicle, S-shape, comma-shaped structures) was determined in LTL-stained tissues in both preterm and term cohorts from 18 to 26 dpc.

We also evaluated the nephrogenic zone using proliferating cell nuclear antigen (PCNA) staining of kidneys from both groups at 23 dpc. Antigen retrieval was performed with 10% citrate buffer and peroxidase quench by hydrogen peroxide in methanol. Sections were blocked in 10% goat serum followed by incubation with a monoclonal PCNA mouse antibody (Invitrogen, 13-3900, 1:100 dilution) overnight at 4 °C then incubated with secondary HRP goat anti-mouse antibody (Bio-Rad, 170-6516, 1:200 dilution) for 1 h. The fixed antibody was detected with DAB.

#### Verification of RNA-Seq data: podocytes (20 dpc), angiogenesis pathway (20 dpc), nephron progenitor cell populations (20–22 dpc) and immune cells (27 dpc)

To identify podocytes, kidney sections from term and preterm animals at 20 dpc were exposed to a 0.45% methanol quench, antigen retrieval with 10% citrate buffer, blockade of endogenous biotin activity with avidin/biotin blocking kit (Vector Laboratories), and 100 μl of goat serum in 1 ml of NaPO_4_ for 1 h. A monoclonal anti-human Wilms Tumor 1 (WT1) mouse antibody (Dako, M3561, 1:100 dilution) was applied to the sections and incubated overnight at 4 °C. The sections were washed in phosphate buffer then incubated with secondary goat anti-mouse antibody (Bio-Rad, 170-6516, 1:200 dilution) for 1 h. Following an avidin–biotin complex to amplify the target antigen signal (Vector Laboratories, Avidin/Biotin Blocking Kit, SP-2001), the fixed antibody was detected with DAB. The sections were counterstained with methylene blue. Ten images were taken just under the capsule containing both mature and immature structures. WT-1^+^ nuclei were counted in mature glomeruli (defined as sphere shaped) and S-shaped bodies in preterm (n = 3) and term (n = 3) groups. Following the same procedure, PECAM-1 was detected using a goat polyclonal antibody (R&D, af3628, 1:250) with a rabbit anti-goat secondary antibody. Lysozyme was detected using a rabbit monoclonal antibody (Abcam, 108508, 1:100) with a goat anti-mouse secondary antibody. Lysozyme expression was measured in two ways and compared between preterm (n = 3/sex) and term groups (n = 3/sex): (1) percentage of glomeruli containing a positive lysozyme cell and (2) percentage of glomeruli with a glomerulotubular junction with lysozyme positive cells. There was no difference between the sexes so they were combined.

To detect Six2 and Jag1 (Jagged 1) on 20 and 22 dpc, kidney tissue sections were blocked with donkey serum in 1 ml of 5% BSA in PBS for 1 h, followed by primary antibody application, and incubated overnight at 4 °C. Both Six2 rabbit polyclonal antibody (Proteintech, 11562, 1:200 dilution) and Jag1 rabbit polyclonal antibody (Santa Cruz, 8303, 1:200 dilution) were co-stained with an anti-cytokeratin mouse antibody (Sigma Aldrich, c2562, 1:200 dilution) to outline the ureteric bud derived structures. Sections were incubated with secondary donkey anti-rabbit and anti-mouse antibodies for 1 h (AlexaFluor 594 and 488, 1:250 dilution). Images were obtained using Microscope Leica Microsystems CMS (Leica DM1000 LED) and DMC6200 digital camera.

#### Glomerular area and density (27 dpc)

Sections were stained with PAS to determine glomerular area and density in both the preterm and term groups at 27 dpc. To measure the glomerular area and density of the kidney sections at 27 dpc, the cortex was segmented from the medulla on PAS-stained sections using Amira software (Thermo Scientific) to measure cortical area. The capillary tuft of each glomerulus was outlined in a midsagittal section using Amira to calculate the glomerular area. Glomerular density was reported as the number of glomeruli/cortical area and was normalized to body weight.

## Results

**Figure 1:**
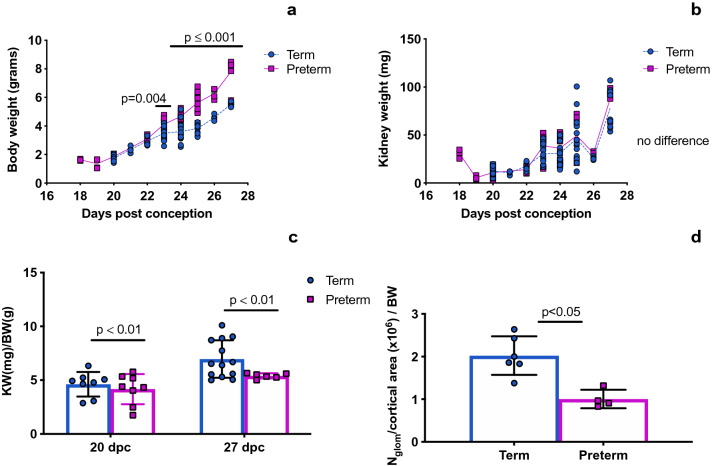
Body weight, kidney weight and glomerular density. Beginning at 23 dpc, the weight of the animals in the preterm group was greater than that of the term group (**a**). There was no difference in the absolute kidney weight between the preterm and term groups (**b**). When kidney weight was normalized to body weight, the preterm group had smaller kidneys at both 20 and 27 dpc (**c**). Glomerular density at 27 dpc was lower in the preterm group than in the term group (**d**). Data was analyzed using a t-test (**a**,**b**) and two-way ANOVA (**c**), Mann–Whitney test (**d**), with a p-value < 0.05 being considered statistically significant.

The body and kidney weights of the preterm and term groups are shown in Fig. 1. The preterm group had a higher body weight than the term group after 23 dpc (Fig. 1a). While absolute kidney weight was similar between the preterm and term groups (Fig. 1b), the kidney weight/body weight (KW/BW) was smaller in the preterm cohort on both 20 and 27 dpc (Fig. 1c). Glomerular density was lower in the preterm group compared to the term group at 27 dpc (Fig. 1d).

### Transcriptional alterations in the kidney are present just after birth in preterm mice

**Figure 2:**
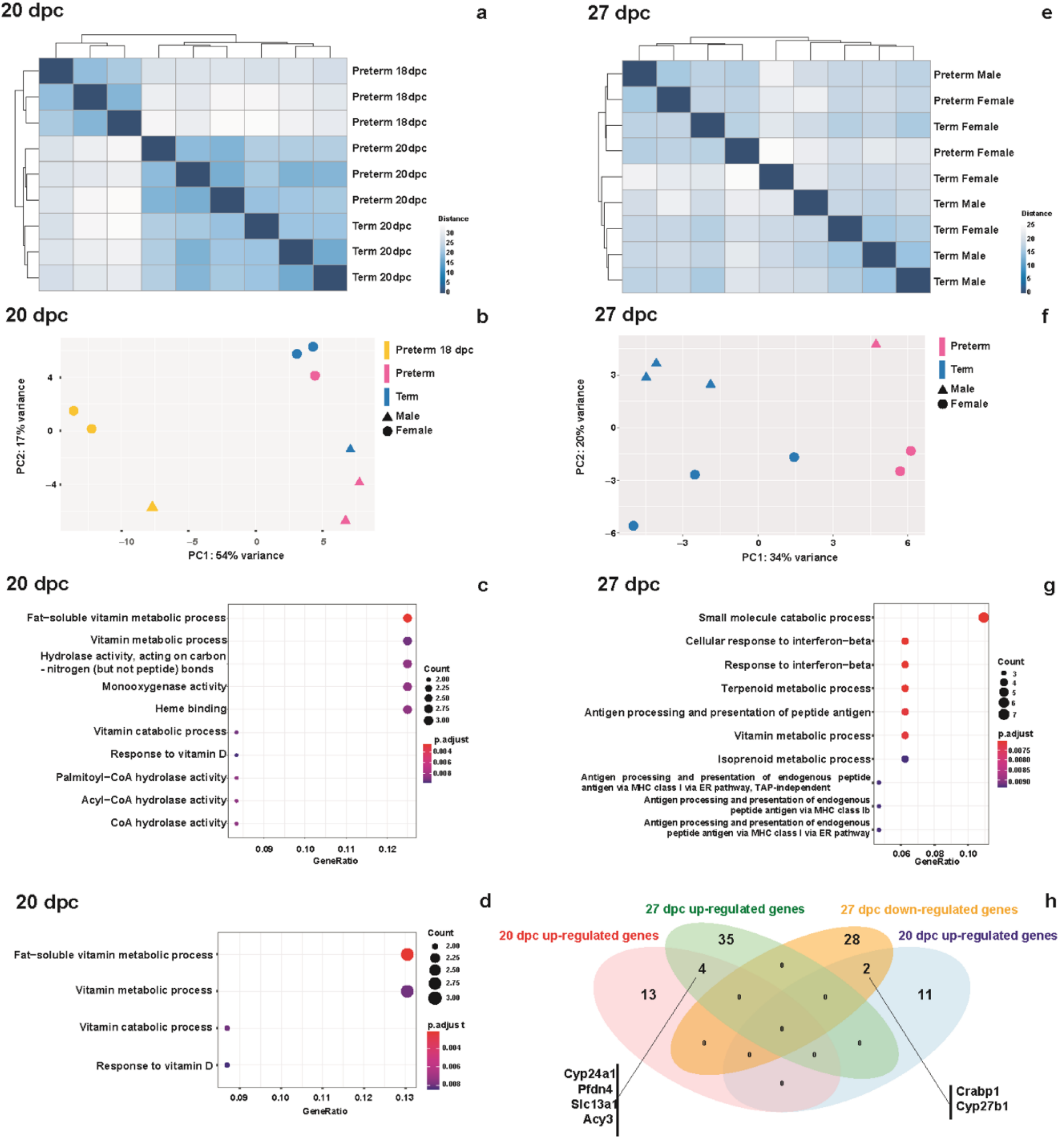
Preterm birth results in early transcriptional alterations in the kidney. (**a**) Hierarchical clustering analysis on RNA-seq data revealed the expression profiles clustering into three groups at the early time point (18 and 20 dpc), with apparent differences between the 18 dpc animals from those at 20 dpc. (**b**) A principal component analysis also showed clear dissociations between 18 and 20 dpc expression profiles. Among the 20 dpc animals, there was sex-dependent clustering. Gene Ontology (GO) enrichment analyses (**c**) and (**d**) on differentially expressed genes (DEGs) between preterm and term 20 kidneys revealed enrichment of the fat-soluble vitamin related pathways in an analysis adjusted (**d**) or not adjusted (**c**) for sex. (**e**) Hierarchical clustering analysis on the expression profiles of 27 dpc animals. (**f**) A principal component analysis of 27 dpc expression data also showed dissociations between preterm and term expression profiles and a sex-dependent clustering. (**g**) GO enrichment analysis on DEGs of 27 dpc without adjusting for sex showed small catabolic processes as the top GO term. (**h**) A Venn diagram showed overlaps of DEGs between 20 and 27 dpc analyses. Six overlapped DEGs were identified (4 up-regulated genes and 2 down-regulated genes) with the same direction of the alterations at 20 and 27 dpc.

Transcriptional alterations were assessed at two time points, (early, 18–20 dpc and later: 27 dpc). We performed an unsupervised analysis of the kidneys at 18 dpc and 20 and 27 dpc in the preterm and term groups. At the early time point (18 and 20 dpc), the expression profiles clustered into three groups (Fig. 2a). The 18 dpc group had distinct gene expression alterations compared to the groups at 20 dpc, as expected. A principal component analysis (PCA) indicated that a sexual dimorphism strongly contributed to PC2 (17% variance) (Fig. 2b). Therefore, we further evaluated the gene expression profiles both with and without adjustment for sex. We identified 39 and 38 differentially expressed genes (DEGs) between preterm and term groups at 20 dpc, 760 and 657 DEGs between 18 dpc and preterm at 20 dpc, and 660 and 583 DEGs between 18 dpc and term at 20 dpc, without and with adjustment for sex. The overlapped DEGs without and with adjustment for sex were 28 (preterm and term at 20 dpc), 607 (18 dpc and preterm at 20 dpc), and 566 (18 dpc and term at 20 dpc), respectively.

The complete lists of DEGs are shown in Supplementary Table S4–S6. To assess the biological functions of the DEGs, we performed gene ontology (GO) enrichment analyses using an R software package, clusterProfiler^37^. The vitamin D-related pathways were enriched in the top GO terms with and without adjusting for sex (Fig. 2c,d), suggesting that the transcriptional alterations occur early. Changes were observed in the preterm group two days after birth.

At 27 dpc, the preterm and term groups did not fully segregate in the hierarchical clustering analysis (Fig. 2e). However, PCA showed a clear distinction between preterm and term in PC1 (34% variance). We observed segregation by sex in PC2 (20% variance) (Fig. 2f), similar to the analysis at 20 dpc. We identified 703 and 558 DEGs between preterm and term kidneys at 27 dpc, without and with adjustment for sex. The complete lists of DEGs in each comparison are shown in Supplementary Table S7. While the number of DEGs was larger than at 20 dpc, there were no significant GO terms after adjusting for sex. Without adjusting for sex, the top GO term in the analysis of DEGs was small molecular catabolic processes (Fig. 2g).

We compared the lists of DEGs between preterm and term kidneys to identify overlapped genes. Four genes (*Cyp24a1*, *Pfdn4*, *Slc34a1*, *Acy3*) were differentially up-regulated at days 20 and 27. Two genes (*Crabp1*, *Cyp27b1*) were differentially down-regulated at both days (Fig. 2h). We confirmed the RNA-Seq gene expression results using qPCR analysis (Supplementary Fig. S2).

### Fewer endothelial cells and podocytes with alterations of nephron progenitor cells in preterm kidneys

**Figure 3:**
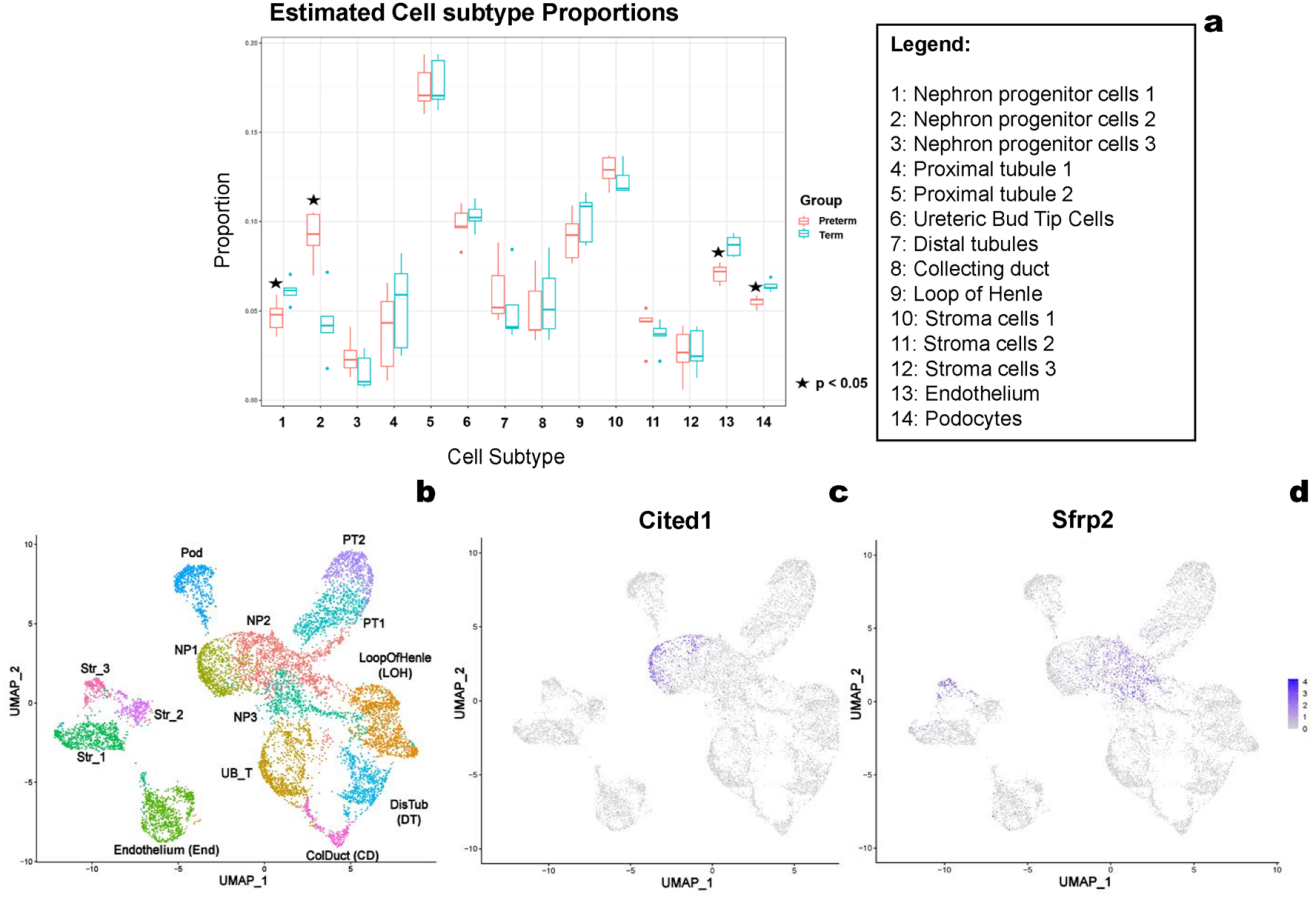
Preterm birth alters the cell subtype proportion of the kidney. A cell subtype proportion analysis on RNA-seq data showed kidneys from the preterm group had a decreased proportion of nephron progenitor 1 cells (NP1) and an increased proportion of NP2 cells (NP). The cell proportion of endothelial cells and podocytes was also lower in the preterm group. Identified cell subtype clusters in the scRNA-seq data (**b**). The nephron progenitor subpopulation marker genes Cited1 and Sfrp1 were used to classified the NP subpopulations with Cited1 marking the nephron progenitor 1 cluster (NP1) (**c**) and Sfrp2 marking the more differentiated nephron progenitor 2 cluster (NP2) (**d**). *Abbreviations in part b: NP1–3, nephron progenitor cells 1–3; UB_T, ureteric bud tip cells; Str_1–3, stromal cells 1–3; Pod, podocytes; PT1–2, proximal tubule 1–2; LOH, Loop of Henle; DT, distal tubule; CD, collecting duct; End, endothelium.

We investigated the relative proportion of cell subtypes in preterm and term groups at 20 dpc using deconvolution of bulk gene expression (Fig. 3a). We found 14 clusters of cell types in the scRNA-seq data, with the expression of 11,845 genes in 9865 cells (Fig. 3b). Both the proportion of endothelial cells and podocytes were lower in the preterm compared to the term group (Fig. 3a). The *Cited1*^+^ nephron progenitors (NP1, Fig. 3c) were decreased, and *Sfrp2*^+^ nephron progenitors (NP2, Fig. 3d) were increased in preterm mice (Fig. 3a). *Sfrp2* is a target of the Wnt4 signaling pathway in kidney development^38^, a key pathway for nephron progenitor differentiation^39^. Together with the premature depletion of Six2 and increased Jag1 expression in the nephrogenic zone, these results suggest that the nephron progenitors prematurely differentiate in the preterm kidney.

### Alterations in angiogenesis and immune cell pathways

**Figure 4:**
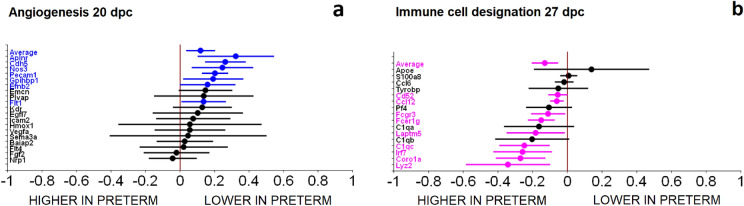
Angiogenesis at 20 dpc and immune cell designation at 27 dpc were the top differential gene expression pathways. At 20 dpc, differential gene expression of seven (gene names highlighted in red) out of 19 genes with confidence intervals (CI) indicating lower gene expression in the preterm group as compared to the term group were observed in the angiogenesis pathway (**a**). At 27 dpc, the category of immune cell designation showed nine of the sixteen genes that had CI’s with higher gene expression in the preterm group compared to the term group (**b**).

The top three differential gene expression pathways at 20 dpc were angiogenesis (Fig. 4a), components of the glomerular basement membrane, and the renin-angiotensin system. Forest plots of all of the pathways are included in Supplementary Fig. S3. The angiogenesis pathway and seven individual genes within the category (*Aplnr, Cdh5, Nos3, Pecam1, Gpihhbp1, Efnb2, Flt1*) had CI’s that indicated lower gene expression in the preterm group compared to the term group (Fig. 4a). In the glomerular basement membrane category, six genes (*Lama5, Col4a6, Fras1, Col4a2m Col4a1, Col4a4*) had CI’s indicating lower expression in the preterm group. The exception was *Lama4* which had a CI indicating higher expression in the preterm group. Three genes (*Anpep, Agtr1-alpha, Cma1*) included in the renin-angiotensin pathway had CI’s that indicated lower expression in the preterm group and three genes (*Nln, Agtr1-beta, Agt*) that had higher expression in the preterm group. None of the differences in individual genes reached statistical significance using the adjusted p < 0.05.

At 27 dpc, the top three pathways of differently expressed genes included immune cell designation (Fig. 4b), proximal tubule, and renin-angiotensin pathway. The category of immune cell designation and nine of the sixteen genes within this pathway had CI’s that indicated higher gene expression in the preterm group (*Cd52, Ccl12, Fcgr3, Fcer1g, Laptm5, C1qc, Irf7, Coro1a, Lyz2*) compared to the term group. However, none of the individual genes reached statistical significance using the adjusted p < 0.05. In the proximal tubule pathway, seven of the 25 genes were differentially expressed. Four genes (*Gsta2, Hdc, Slc34a1, Pdzk1*) had CI’s indicating lower expression in the preterm group and three genes (*Slc5a2, Slc5a1, Slc3a1*) had CI’s indicating higher expression in the preterm group (Supplementary Fig. S3). The genes *Pdzk1*, *Gsta2*, and *Slc34a1*, were significantly lower in the preterm group compared to the term group, using the adjusted p < 0.05 (Table 1). The renin-angiotensin pathway had six differentially expressed genes, with three genes with a CI indicating a lower expression in the preterm group (*Anpep, Enpep*, and *Ace2*), and three genes had CI indicating higher expression in the preterm group (*Agtr1-beta, Ren1 and Ren2*). *Ren1*, and *Ren2* were significantly greater in the preterm group using the adjusted p < 0.05 (Table 1).

**Table 1:** Differentially expressed genes at 27 dpc.

Gene symbol	Estimate (higher in preterm mice)	Standard error	95% confidence interval	p-value
*Rgs5*	− 0.272	0.039	(− 0.365, − 0.179)	0.0002*
*Ren2*	− 0.445	0.067	(− 0.603, − 0.287)	0.0003*
*Ren1*	− 0.445	0.067	(− 0.603, − 0.287)	0.0003*
*Etv4*	− 0.283	0.044	(− 0.388, − 0.178)	0.0004*
*Slc5a2*	− 0.175	0.028	(− 0.242, − 0.108)	0.0004*
*Etv5*	− 0.307	0.052	(− 0.430, − 0.183)	0.0006*
*Mgp*	− 0.522	0.089	(− 0.732, − 0.312)	0.0006*

Gene symbol	Estimate (lower in preterm mice)	Standard error	95% Confidence Interval	p-value
*Pdzk1*	0.293	0.029	(0.224, 0.361)	< 0.0001 *
*Gsta2*	0.879	0.101	(0.639, 1.118)	0.0001*
*Slc34a1*	0.330	0.049	(0.214, 0.447)	0.0003*
*Igf2*	0.503	0.086	(0.299, 0.707)	0.0006*
*Cldn5*	0.214	0.039	(0.123, 0.306)	0.0009*
*Wnt11*	0.278	0.056	(0.146, 0.410)	0.0016*

The p values and confidence intervals were based on the two-sample t-test with pooled variance with an adjust p value < 0.05.

Podocyte gene expression was examined at 20 and 27 dpc because deconvolution analysis showed fewer podocytes at 20 dpc. At 20 dpc, four genes had CI’s indicating a lower expression in the preterm group (*Nphs1, Plce1, Lmx1b, and Magi2*). At 27 dpc, four genes had a CI indicating a lower expression in the preterm group (*WT1, Hs3st6, Plce1, and Foxd2*). However, differential expression of these genes did not reach the threshold for significance.

### Premature cessation and differentiation of nephron progenitors following preterm birth

**Figure 5:**
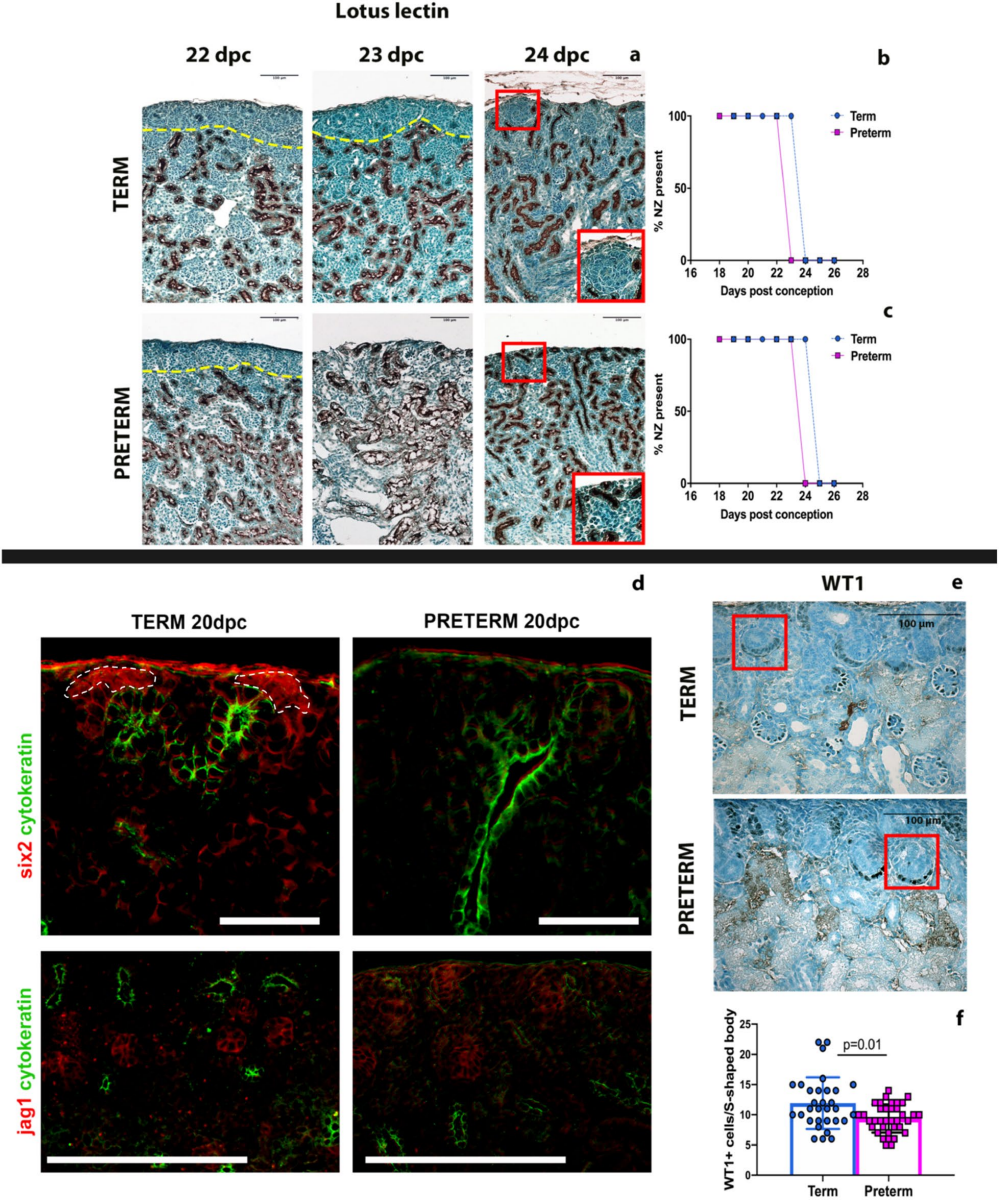
Preterm mice have a shorter duration of nephrogenesis. The nephrogenic zone is outlined by the yellow line in the LTL-stained kidneys (**a**). The nephrogenic zone was depleted when there were no longer cap mesenchymal cells and when LTL + cells reach the top of the capsule. The nephrogenic zone is absent in the preterm cohort one day before the term group (**b**). The same LTL-stained tissue was used to determine the presence of immature structures (vesicles, comma- and S-shaped bodies), which were not detectable in preterm animals one day before than in the term cohort (**c**). In the red square in panel a of a term mouse kidney at 24 dpc, the S-shaped body indicates the presence of immature structures at this time point in the term group. However, the preterm group lacks these immature structures. Representative pictures of Six2 and Jag1 staining showing the loss of the Six2 protein expression in the nephrogenic zone and increase of the Jag1 expressed in the nephrogenic zone, consistent with early differentiation of the preterm group at 20 dpc (**d**). Panel (**e**) is a representative image of WT-1 staining within the kidney of a term and preterm mouse at 20 dpc. Within the red box, eight WT-1^+^ cells can be counted within the s-shaped body, whereas, in panel e, there are only 6 WT1^+^ cells. Interestingly, under identical conditions, the WT1^+^ cells consistently appeared darker in the preterm group as compared to the term group, which may be related to a more advanced state of differentiation. Overall, the term group had more WT1^+^ cells in s-shaped bodies than the preterm group at 20 dpc (**f**). Data was analyzed using a Mann–Whitney test, with a p-value < 0.05 being considered statistically significant. Scale bars in (**a**) and (**e**) = 100 µm. Scale bars in d: Six2/cytokeratin = 100 µm; in Jag1/cytokeratin = 200 µm.

We identified immature nephron structures using the LTL-stained tissue samples to assess postnatal nephrogenesis. During nephrogenesis, the nephrogenic zone was present until 23 dpc in the preterm group and until 24 dpc in the term group (Fig. 5a,b). After the cap mesenchymal cells were no longer present, the immature structures including vesicles, comma-shaped bodies, and S-shaped bodies, remained identifiable until 24 dpc in the preterm group and 25 dpc in the term group (Fig. 5c). There was ongoing proliferation in the nephrogenic zone of both groups highlighted by PCNA staining at 23 dpc. However, proliferation in the location of the cap mesenchyme was absent and the nephrogenic zone was narrower in the preterm group (Supplementary Fig. S4). The progenitor cell population was further characterized by the histologic expression of *Six2* and *Jag1* in the nephrogenic zone. *Six2*^+^ cells were present in the cap mesenchyme in the term group at 20 dpc, but were absent in the preterm group at 20 dpc. There was no expression of *Six2* in the cap mesenchyme of either the term or preterm groups at 22 dpc. *Jag1* was expressed in the preterm and term groups at 20 and 22 dpc, but the location of the *Jag1* was more prominent just under the capsule in the preterm group, where the cap mesenchyme had been depleted (Fig. 5d). Together, these data indicate that animals born preterm have premature termination of nephrogenesis. In addition, there were fewer WT1 + cells in S-shaped bodies in the preterm group than in the term group (Fig. 5e,f).

To verify the decreased expression of genes involved in angiogenic pathway and increased expression of genes in the immune cell pathways of the preterm group, we examined the kidney tissue at 20 and 27 dpc in each group. At 20 dpc, PECAM-1 was expressed in the glomeruli and peritubular capillaries. However, the expression of PECAM-1 appeared to be more highly expressed in the mature glomeruli of the term group as compared to the preterm group. At 27 dpc in the preterm group, lysozyme was observed more commonly in the glomeruli and at the junction of the glomerulotubular connection (Supplementary Fig. S5).

## Discussion

In this study, we demonstrate that preterm birth in mice is sufficient to induce early cessation of nephrogenesis by premature differentiation of the nephron progenitor cells. In an unbiased analysis, we observed dysregulation of the expression of genes related to fat-soluble vitamin metabolism at both 20 and 27 dpc. In a candidate gene approach, the pathway of angiogenesis was downregulated at 20 dpc and immune cells were upregulated at 27 dpc in the preterm mice. Importantly, preterm birth alone alters the expression of genes related to proximal tubules and involved in the renin-angiotensin pathway. Mice born preterm emulate many of the characteristics observed after preterm birth in humans, and the preterm mouse should serve as a model to dissect the molecular mechanisms connecting preterm birth to kidney disease and hypertension.

In humans, nearly 60% of nephrogenesis occurs in the third trimester^40,41^. Preterm birth in humans leads to early cessation of nephrogenesis and accelerated maturation of nephrons. Little is known about the fate of the progenitor pool, the signals that determine cessation of nephrogenesis, or why the duration of nephrogenesis varies between species. Hartman et al^42^ reported a consistent end to murine nephrogenesis at 24 dpc in CD-1 mice. In the current study, the cap mesenchyme was consistently depleted by 23 dpc in the preterm group and by 24 dpc in the term group, indicating early cessation of nephrogenesis in the preterm group by one day earlier than in the term group. A loss of one day of nephrogenesis is significant in mice because nearly 20% of glomeruli are created on the last day of nephrogenesis^43^. The premature end of nephrogenesis and lower glomerular density in the preterm group at 27 dpc in this study are also consistent with the original findings of this model published by Stelloh et al.^18^, which showed a 24% lower glomerular number in the preterm group.

There are several mechanisms that could lead to the premature cessation of nephrogenesis in this model, including the loss or premature differentiation of the self-renewing population of progenitor cells or decreased branching of the ureteric bud. Although reduced branching of the ureteric bud can reduce nephron number, this is less likely playing a role in this model because the change in environment is late in gestation, when the majority of branching is completed^44^. Our data support premature differentiation of the self-renewing population of progenitor cells as the primary mechanism leading to early cessation of nephrogenesis. We observed the loss of Six2 and an altered location of Jag1 protein expression in the nephrogenic zone of the preterm group at 20dpc. The NP1 cell population was also significantly lower in the preterm kidney, but the NP2 population was higher. The NP1 population expressed the highest level of *Cited1* (Fig. 3c) and *Six2* (Supplementary Fig. S6). The NP2 population had a lower expression of *Cited1* (Fig. 3c), but retained *Six2* expression (Supplementary Fig. S6) with a higher expression of *Lhx1*, *Jag1*, and *Sfrp2*, indicating a more differentiated cell type in NP2. Using pseudotime trajectory analysis, we further examined these changes in the populations (Supplementary Fig. S7). Most cells in the NP1 population that decreased in the preterm were in G1 of the cell cycle. Most cells in the NP2 population were in S or G2, suggesting they were proliferating (Supplementary Table S8). Together, this suggests that these NP1 cells may represent an early progenitor population with a slow turnover (*Cited1*^+^, *Six2*^+^), while NP2 may represent a more differentiated but proliferating population (*Cited1*^−^, *Six2*^+^, *Sfrp2*^+^), as previously described^39,45,46^. The pseudo-trajectory analysis also suggested that the NP2 population represents nephron progenitor cells between NP1 and more differentiated cells (Supplementary Fig. S7). Together, these data suggest preterm birth leads to depletion of multipotent (*Cited1*^+^, *Six2*^+^) progenitor cells, leading to early nephron cessation. Further work is necessary to understand the signaling that leads to premature differentiation of the nephron progenitors, including the metabolic factors, changes in stromal signaling, or alterations in the epigenetic regulation of these cells after birth.

We also identified alterations in several key kidney developmental pathways in the preterm group at 20 dpc, including perturbations in angiogenesis and lower endothelial cell proportions. Several genes (*Ren1*, *Ren2*) in the renin-angiotensin system were also higher in the preterm group at 27 dpc. *Ren1* encodes for renin, which cleaves angiotensinogen to angiotensin 1 critical in blood pressure control. Angiotensin 1 is cleaved by Angiotensin-Converting Enzyme to Angiotensin II. Angiotensin II causes vasoconstriction and increases secretion of antidiuretic hormone and aldosterone, leading to increased blood pressure. These findings in the preterm mice kidney are consistent with known complications of higher blood pressures and hypertension in humans born preterm^19,20,47,48^. Infants born preterm have high levels of renin in serum plasma^49^, and many develop neonatal hypertension. The role of disrupted angiogenesis within the kidney is also consistent with studies in preterm baboons, which indicated poor glomerular vascularization^22^.

Using a publicly available single-cell sequencing dataset, we also observed a decrease in the subpopulation of podocytes. This finding of a smaller subpopulation of podocytes at 20 dpc in the preterm group is consistent with human and animal studies. A limited study of biopsies of adolescents and adults who were born preterm demonstrates decreased podocyte density and FSGS lesions^50^. Indeed, the urine of preterm neonates is a novel source for progenitor cells, with a higher number of podocytes in the urine of infants being treated with nonsteroidal anti-inflammatory drugs^51,52^. The podocytes in the superficial cortex of the preterm baboon exhibit an abnormal, immature pattern of WT1 + cells surrounding relatively undifferentiated cells^22^. Our findings that preterm birth is associated with fewer podocytes per glomerulus and a disorganized pattern of WT1^+^ cells in the immature bodies in the nephrogenic zone suggest a podocytopathy may underlie the transition from preterm birth to CKD.

In our unbiased analysis approach, the fat-soluble vitamin pathway was altered; *Cyp27b1* was downregulated in the preterm group, but *Cyp24a1* was upregulated. *Cyp27b1* and *Cyp24a1* encode members of the cytochrome P450 superfamily of enzymes that play a role in vitamin D metabolism. *Cyp27b1* encodes for the enzyme 1a-hydroxylase, increasing activated vitamin D. *Cyp24a1* encodes for 24-hydroxylase, which inactivates vitamin D. Downregulation of 1a-hydroxylase and upregulation of 24-hydroxylase in our preterm group indicates a reduced level of biologically active vitamin D. Vitamin D has a significant role in organogenesis; it plays a role in bone metabolism and maintenance of calcium and phosphorus homeostasis, and has broad functions in cardiovascular health, autoimmune response, and insulin secretion^53–56^. Vitamin D deficiency is prevalent in preterm neonates because the fetus cannot produce vitamin D. In preterm neonates, vitamin D deficiency has been associated with respiratory distress syndrome^57^ and predicts respiratory morbidity^58^. Although there is little known about the effect of vitamin D deficiency on kidney development, clinical studies indicate a relationship. In a nested study within the Generation R study, investigators demonstrate a correlation between higher maternal 25-hydroxyvitamin D and lower childhood estimated glomerular filtration rate^59^. In preclinical studies in rodents, supplementation of vitamin D appears to reduce nephrogenesis, suggesting a role for vitamin D-dependent suppression of angiotensin II. Maternal vitamin D deficiency is associated with delayed glomerular maturation^60^, but it is unclear if these nephrons have any functional impairment. A delicate balance of vitamin D activity is likely needed for nephrogenesis, and further work is required to define the role of vitamin D in the overall kidney health of preterm neonates.

Another perturbation of the fat-soluble vitamin pathway in the preterm group was the downregulation of *Crabp1* (encoding for cellular retinoic acid-binding protein-1). Vitamin A regulation is complex. The majority of the biologic functions^61^ of this pathway are accomplished by retinoic acid. There is evidence that upregulation of *Crabp1* can lower intracellular retinoic acid concentrations to reduce differentiation of the cell, modulate the metabolism of retinoic acid to 4-oxorentinoic acid, and increase the movement of the retinoic acid to the nucleus to interact with retinoic acid receptor initiating gene transcription^62,63^. The downregulation of *Crabp1* in the preterm mice may cause decreased retinol signaling and contribute to decreased progenitor cell self-renewal^64^. The vitamin A pathway also has an impact on kidney development. There is a clear role of retinol deficiency in the developing kidney and a strong correlation between plasma retinol levels and nephron number. Maternal vitamin A deficiency has been associated with renal hypoplasia^65,66^. Retinoic acid acts on mesenchymal cells to release branching morphogens and induce tubulogenesis^67–71^. Supplementation of vitamin A has also been associated with an increased glomerular number in animal models of intrauterine growth restriction. Beyond the kidney, the vitamin A pathway plays an essential role in organogenesis and development, with significant deficiencies leading to fetal death and multiple congenital disabilities^72^. Similar to vitamin D, vitamin A deficiency has also been reported to be common in preterm neonates. Human and mice studies have also shown the association between vitamin A deficiency and poor lung outcomes^73,74^. Supplementation with vitamin A improved lung outcomes in these preterm neonates^75^.

This study uncovered a few unique perturbations in genes associated with proximal tubule, including *Pdzk11*, *Gsta2*, and *Slc34a1*, which were significantly lower in the preterm group compared to the term group at 27 dpc. Both *Pdzk1* and *Slc34a1* are important in phosphorus reabsorption. *Pdzk1* encodes for a scaffolding protein for renal apical transporters such as uric acid and phosphorus reabsorption^76^. *Slc34a1* encodes for a sodium-dependent phosphate transporter in the brush border of the proximal tubule. *Gsta2* encodes for an enzyme glutathione S-transferase A2 that is involved in the detoxification of electrophilic compounds. The decreased expression of these genes may also play a role in the development of acute kidney injury (AKI). In a murine model of sepsis, down-regulation of sodium phosphate type 2a transporter in the proximal tubule correlated with evidence of AKI^77^. Severe preterm birth is commonly associated with AKI in preterm neonates^8^, and most preterm neonates are exposed to proximal tubule-specific nephrotoxins such as gentamicin^52^. Interestingly, variants of *Slc34a1* have been associated with chronic kidney disease in genome-wide association studies^78^.

The pathway comprised of immune cells was enriched in the preterm group compared to the term group. We confirmed that the lysozyme protein was detected more frequently in the glomeruli and glomerulotubular junction of the preterm group. Previous studies have demonstrated global alterations in both innate and adaptive immune cell populations in preterm rats^79,80^ and pigs^81–83^. Recently a preterm pig model exposed to endotoxin demonstrated immune activation in the kidney^84^. In that study, *Lyz1* expression in the kidney was higher in the preterm group as compared to the term group. Along with higher expression of *LTF*, *S100A9*, the authors concluded that lipopolysaccharide stimulates the innate inflammatory response in the kidney. However, there was no term group included in that piglet study. Therefore, it was not possible to determine if some of the immune response was a result of the preterm birth, as indicated in our study. Further work is necessary to determine if these immune alterations directly impact future pathology in the glomeruli or glomerulotubular junction.

There are limitations of this study, including the availability of scRNA-seq data of neonatal mouse kidneys. Testing expression profile alterations on each nephron progenitor subpopulation using scRNA-seq could provide further insights to understand the effect of preterm birth on kidney development. Sex was only accounted for in the RNA-Seq data in this study. Determination of sex based on anogenital length at 20 dpc is subjective and not reliable at postnatal ages of less than 5 days^85^. Although we did not separate the cohorts by sex in the histologic evaluation, we did query the RNA dataset for gene expression differences in early markers such as *Cited1*, *Six2* and *Jag1* and demonstrated no difference. An additional limitation was the lack of an unbiased metric of glomerular number. Here, we used glomerular density to verify the phenotype of the preterm model, while conserving the small amount of kidney tissue available. Glomerular density is an imperfect surrogate for glomerular number. Although we report a larger percent difference in glomerular density than the percent difference in glomerular density reported by Stelloh et al., glomerular number measured by acid maceration measures all glomeruli, while glomerular density from histologic sections measures only a fraction of them and is sensitive to intra-kidney variability in glomerular density. Finally, only the preterm group was fostered in this study. Although this may introduce a source of bias, there were no differences seen in the Stelloh’s publication^18^ in the C-section and fostered group as compared to the vaginal delivery and no fostering groups. Overall, this model of preterm birth was heterogeneous, with large ranges in several metrics such as kidney weights. Further work is necessary to understand the mechanisms that may lead to these variable responses and outcomes.

Together, our data provide the first insights into gene dysregulation and changes in proportions of cell subtypes that may contribute to kidney disease and risk of hypertension in those born preterm. The preterm mouse model provides a means to dissect the molecular mechanism underlying premature cessation of nephrogenesis to develop targeted strategies to reduce the risk of CKD. This environmental model is unique because the nephrotoxin exposures and other insults common in preterm humans are absent, isolating the effect of preterm birth. Our study demonstrates dysregulation of potentially modifiable pathways in the preterm kidney, including alterations in vitamin metabolism. Further work is required to decipher the molecular mechanisms that influence the cessation of nephrogenesis to safely apply new tools to extend the duration of nephrogenesis in preterm neonates.

## Supplementary Information

Supplementary Tables.

Supplementary Figure S1.

Supplementary Figure S2.

Supplementary Figure S3.

Supplementary Figure S3.

Supplementary Figure S4.

Supplementary Figure S5.

Supplementary Figure S6.

Supplementary Figure S7.
