# Supplementary material for: Premature differentiation of nephron progenitor cell and dysregulation of gene pathways critical to kidney development in a model of preterm birth
Source: Sci Rep. 2021 Nov 4;11:21667. doi: 10.1038/s41598-021-00489-y (PMC8569166; doi:10.1038/s41598-021-00489-y)
Supplement: Supplementary file 2 — Supplementary Figure S1. [file 41598_2021_489_MOESM2_ESM.docx]

**Supplementary Data: Figure S1**

**Premature differentiation of nephron progenitors and dysregulation of gene pathways critical to kidney development in a model of preterm birth**

Aleksandra Cwiek^1^, Masako Suzuki^3^, Kim deRonde^1^, Mark Conaway^4 5^, Kevin M. Bennett^6^, Samir El Dahr^7^, Kimberly Reidy^2#^, Jennifer R Charlton^1#^*


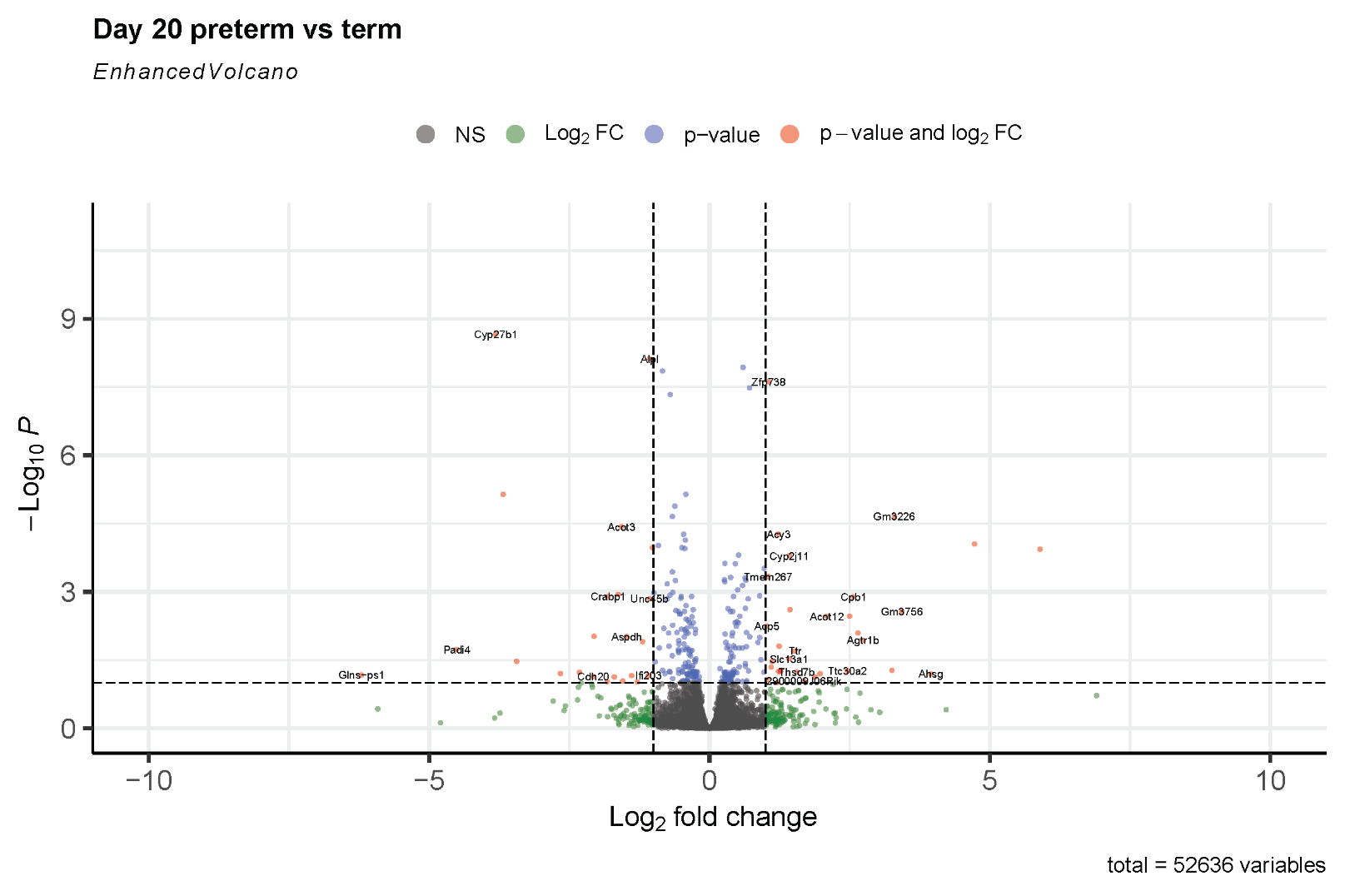


**Supplementary Figure S1a.** Volcano plot generated from RNA-seq data without adjusted for sex, presents the statistical significance (FDR-adjusted p-value) versus the magnitude of change (log2-fold change). Red dots indicate differentially expressed transcripts, and gene symbols were indicated if the transcripts were annotated.


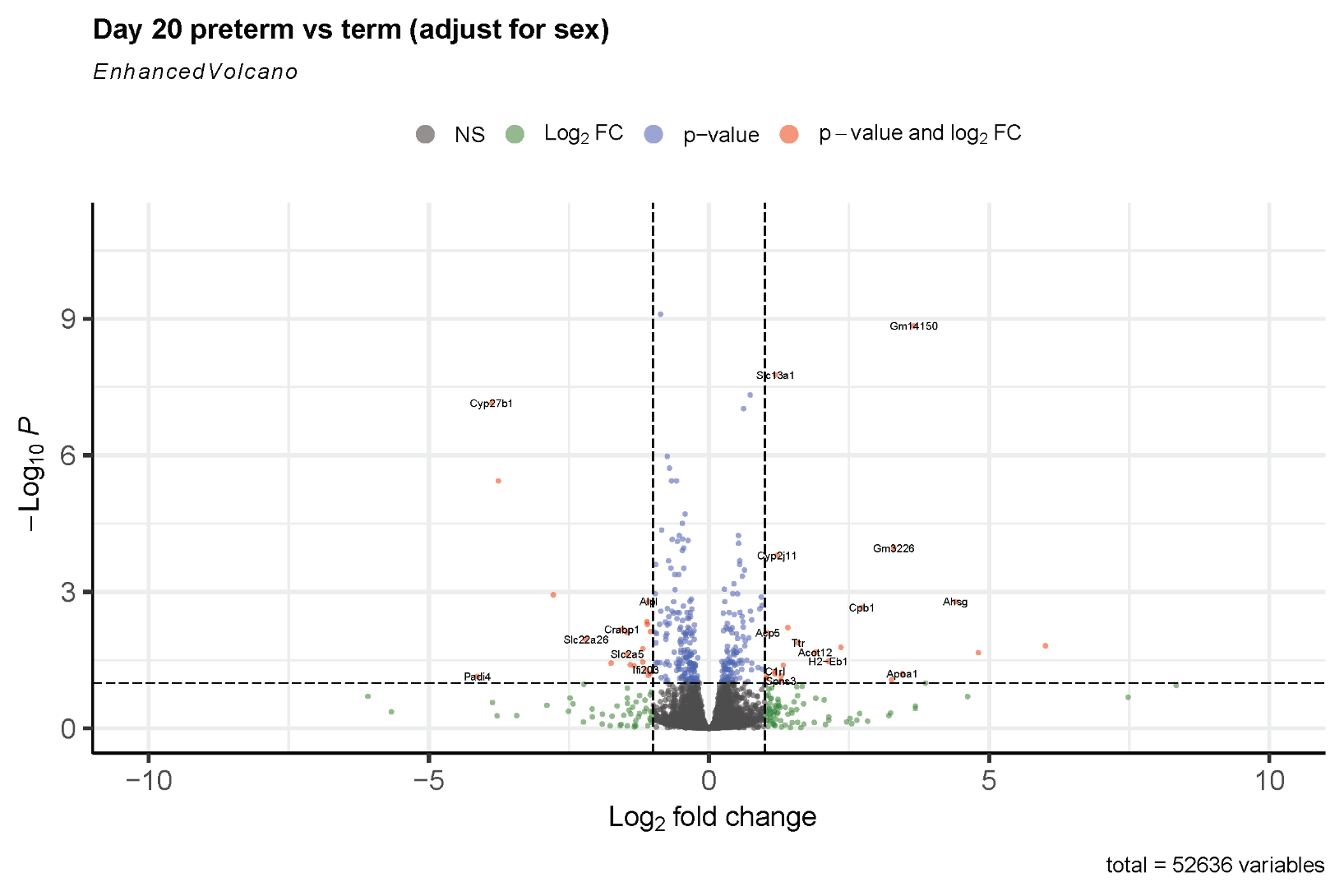


**Supplementary Figure S1b.** The volcano plot was generated from RNA-seq data adjusted for sex, presenting the statistical significance (FDR-adjusted p-value) versus the magnitude of change (log2-fold change). Red dots indicate differentially expressed transcripts, and gene symbols were indicated if the transcripts were annotated.
