# Supplementary material for: Premature differentiation of nephron progenitor cell and dysregulation of gene pathways critical to kidney development in a model of preterm birth
Source: Sci Rep. 2021 Nov 4;11:21667. doi: 10.1038/s41598-021-00489-y (PMC8569166; doi:10.1038/s41598-021-00489-y)
Supplement: Supplementary file 3 — Supplementary Figure S2. [file 41598_2021_489_MOESM3_ESM.docx]

**Supplementary Data: Figure S2**

**Premature differentiation of nephron progenitors and dysregulation of gene pathways critical to kidney development in a model of preterm birth**

Aleksandra Cwiek^1^, Masako Suzuki^3^, Kim deRonde^1^, Mark Conaway^4 5^, Kevin M. Bennett^6^, Samir El Dahr^7^, Kimberly Reidy^2#^, Jennifer R Charlton^1#^*


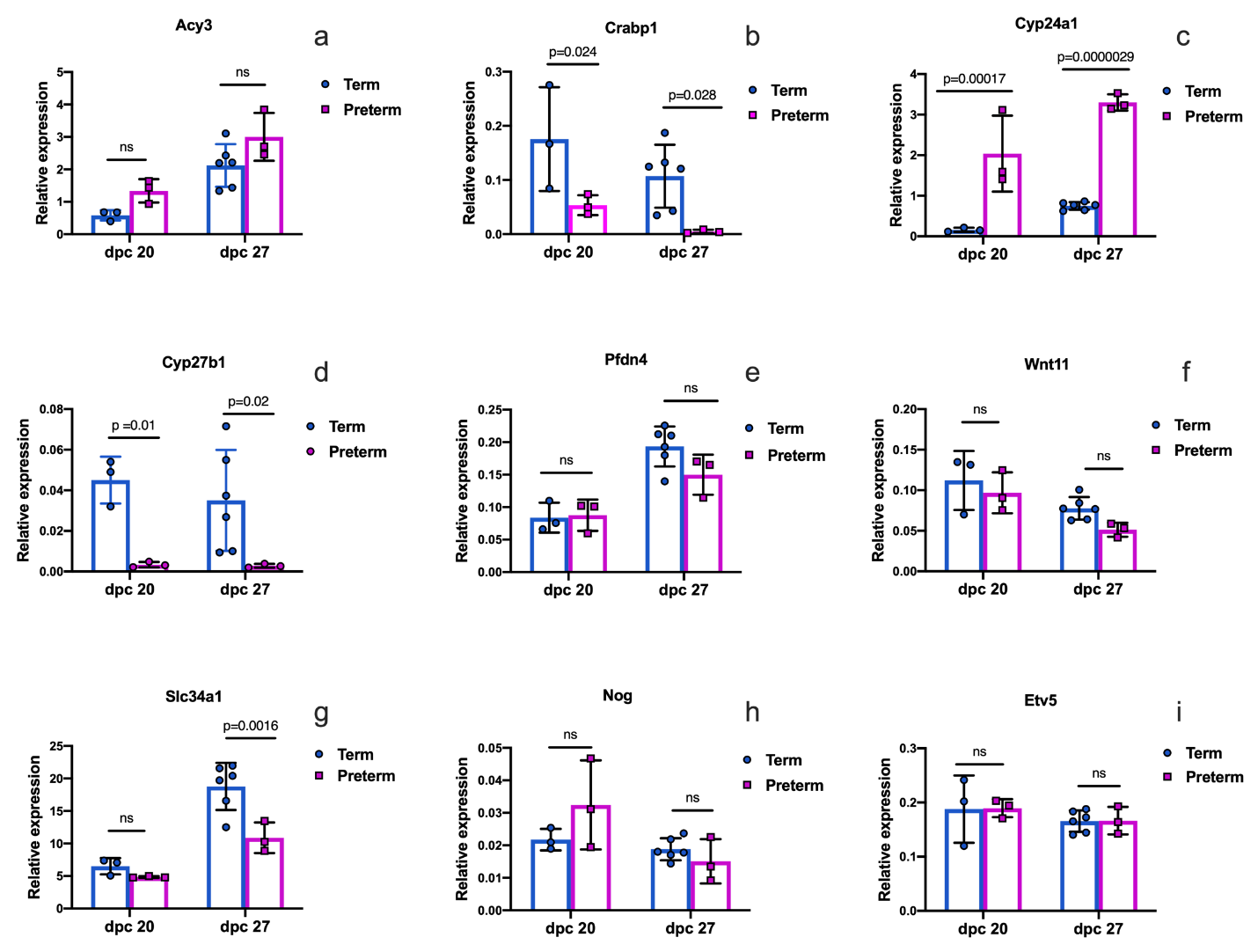


**Supplementary Figure S2.** **Validation of RNA-Seq data using RT-PCR**. RT-PCR analysis was performed to validate RNA-seq results. Relative expression was compared using t-test.
