# Supplementary material for: Premature differentiation of nephron progenitor cell and dysregulation of gene pathways critical to kidney development in a model of preterm birth
Source: Sci Rep. 2021 Nov 4;11:21667. doi: 10.1038/s41598-021-00489-y (PMC8569166; doi:10.1038/s41598-021-00489-y)
Supplement: Supplementary file 4 — Supplementary Figure S3. [file 41598_2021_489_MOESM4_ESM.pdf]

Supplementary Data: Figure S3a

Premature differentiation of nephron progenitors and dysregulation of gene pathways critical to kidney development in a model of preterm birth

Aleksandra Cwiek[1], Masako Suzuki[3], Kim deRonde[1], Mark Conaway[4 5], Kevin M. Bennett[6], Samir El Dahr[7], Kimberly Reidy[2#], Jennifer R Charlton[1#]*

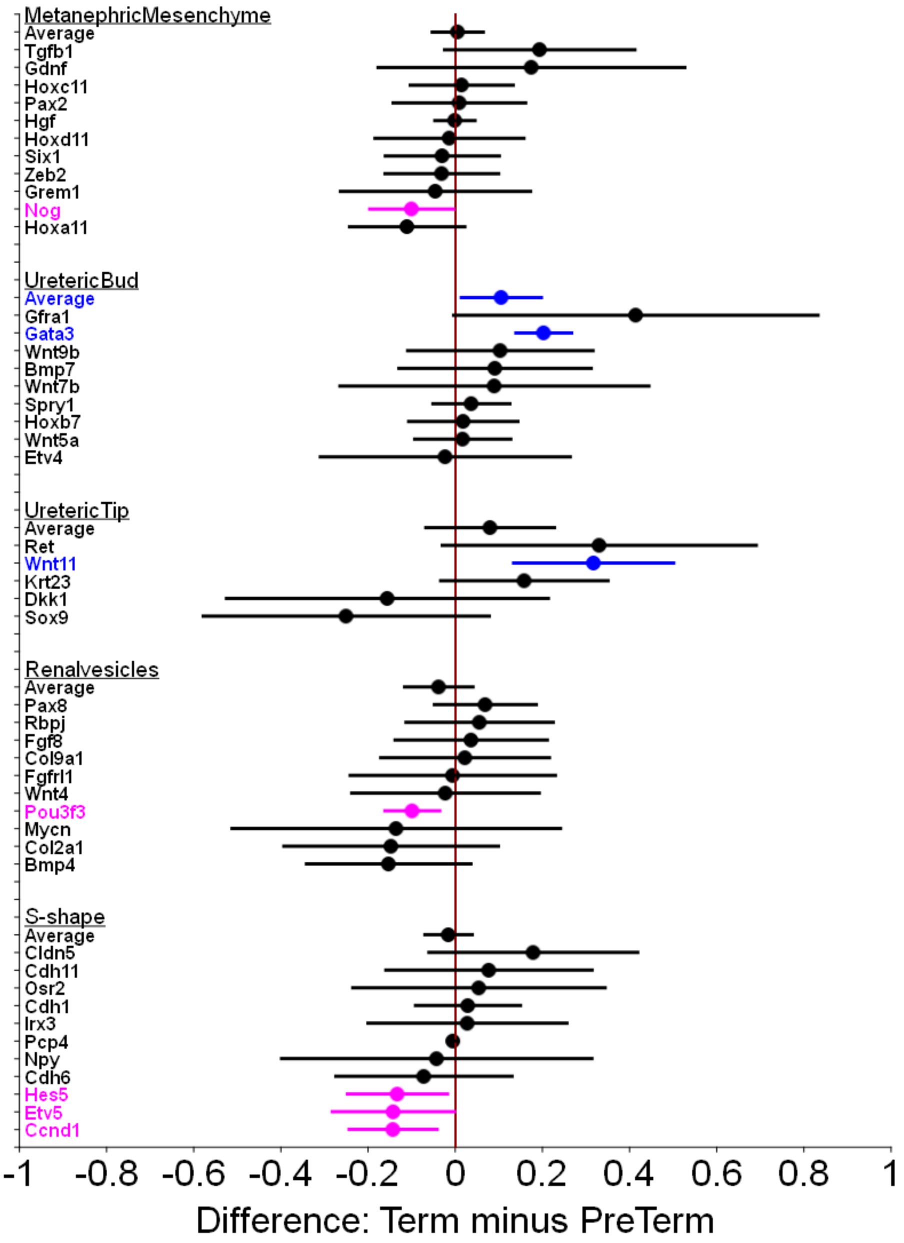

Supplementary Figure S3a. Kidney development category at 20 dpc.

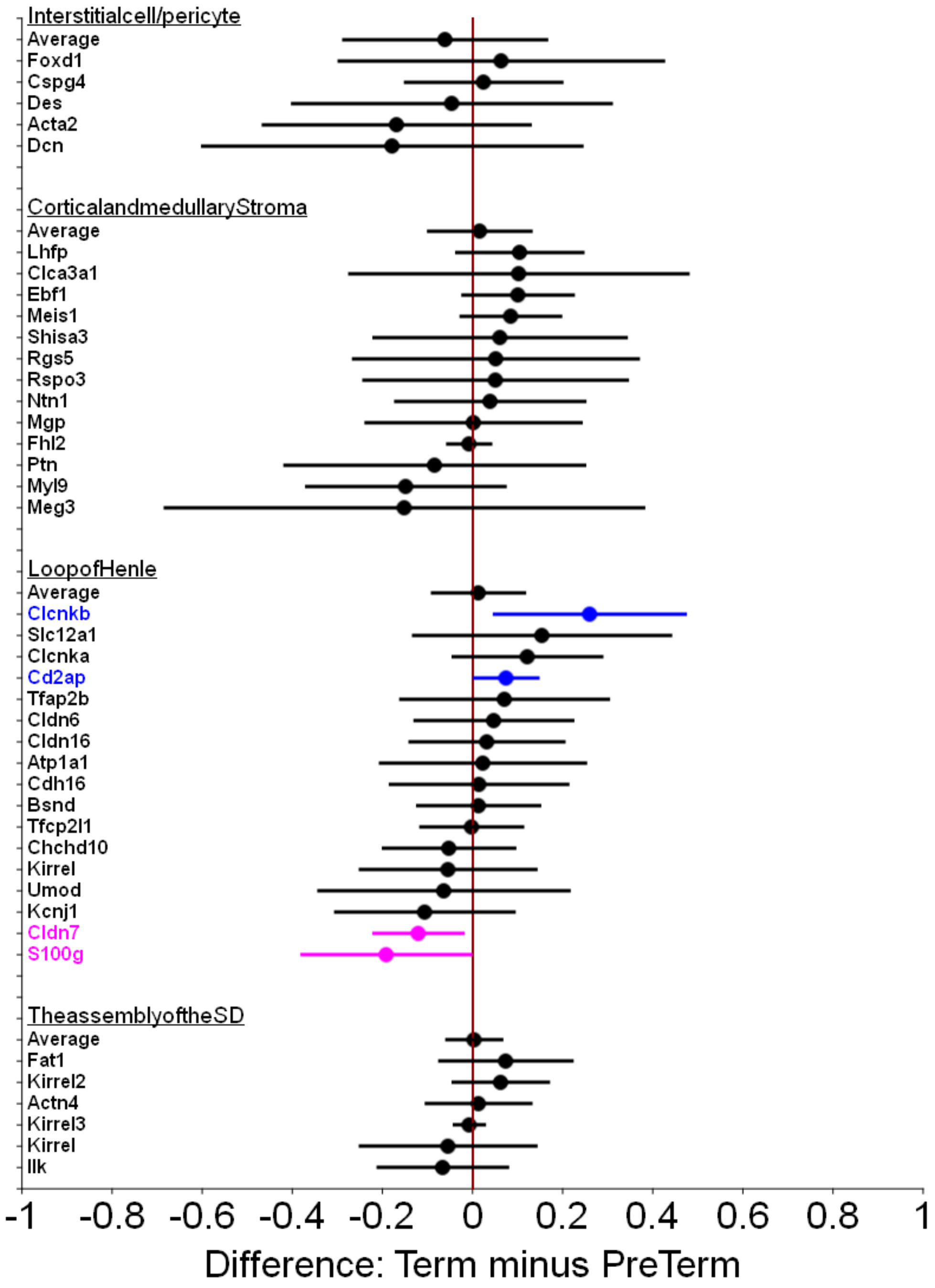

Supplementary Figure S3a. Nephron structure category part 1 of 3 at 20 dpc.

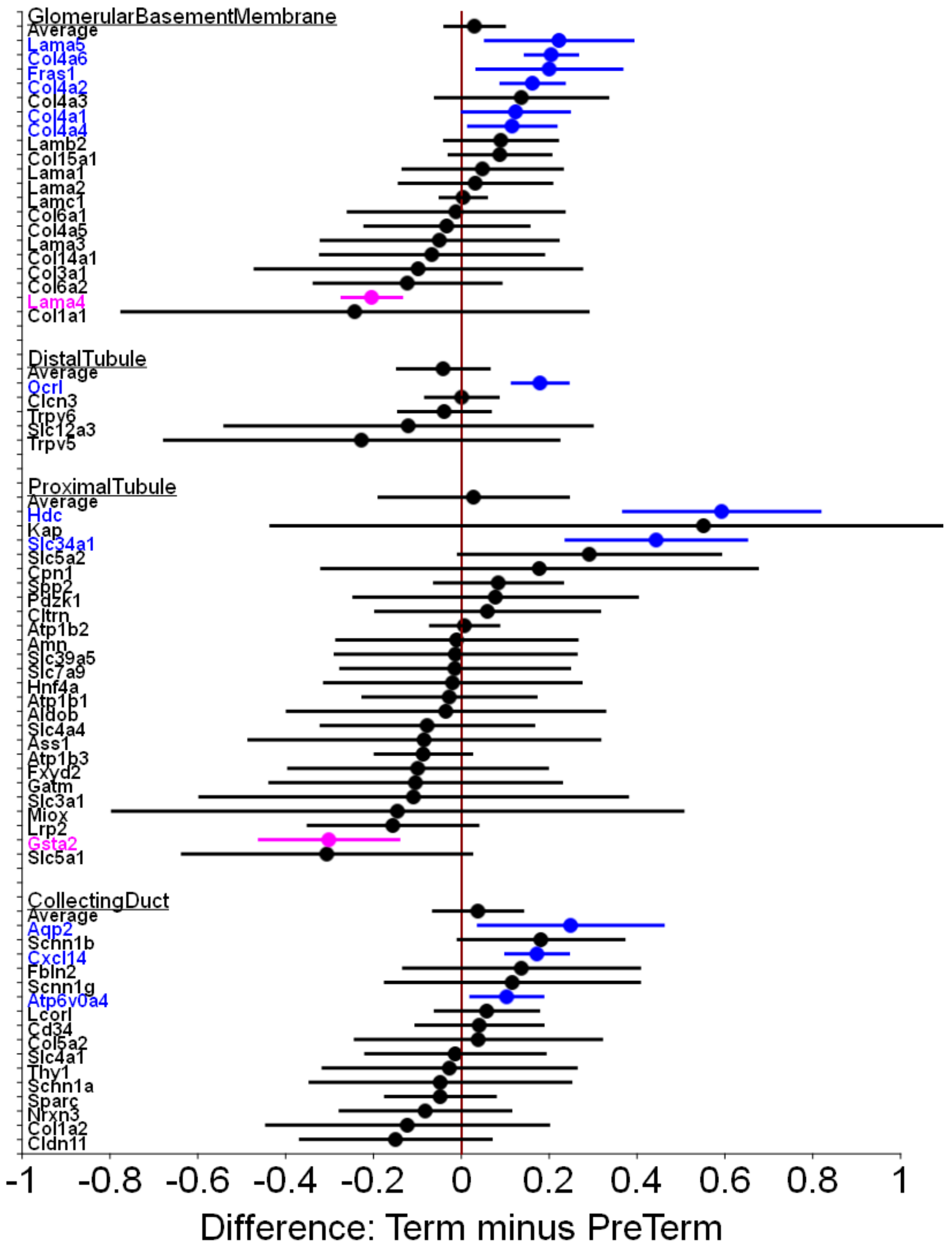

Supplementary Fig. S3a. Nephron structure category part 2 of 3 at 20 dpc.

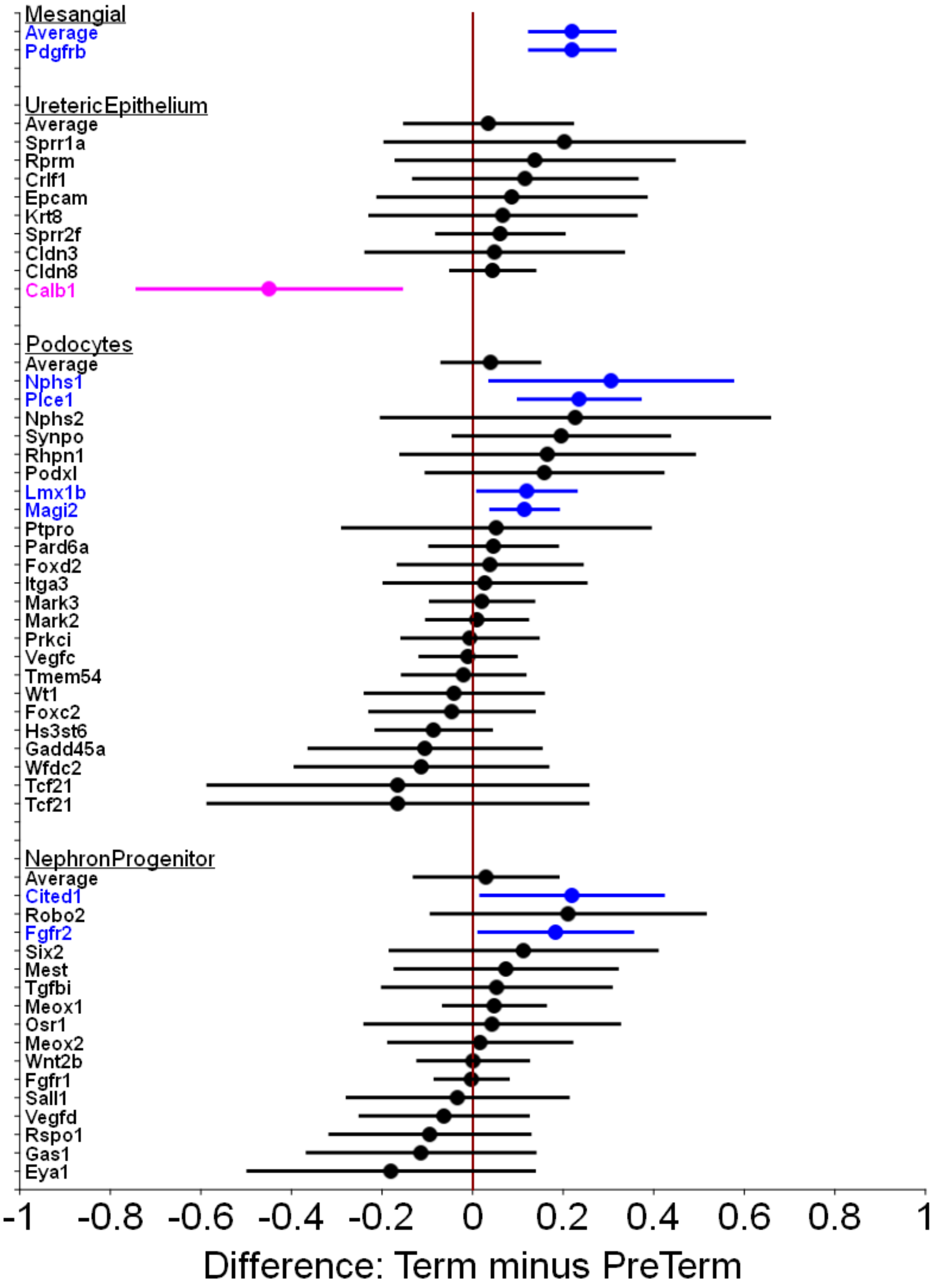

Supplementary Figure S3a. Nephron structure category part 3 of 3 at 20 dpc.

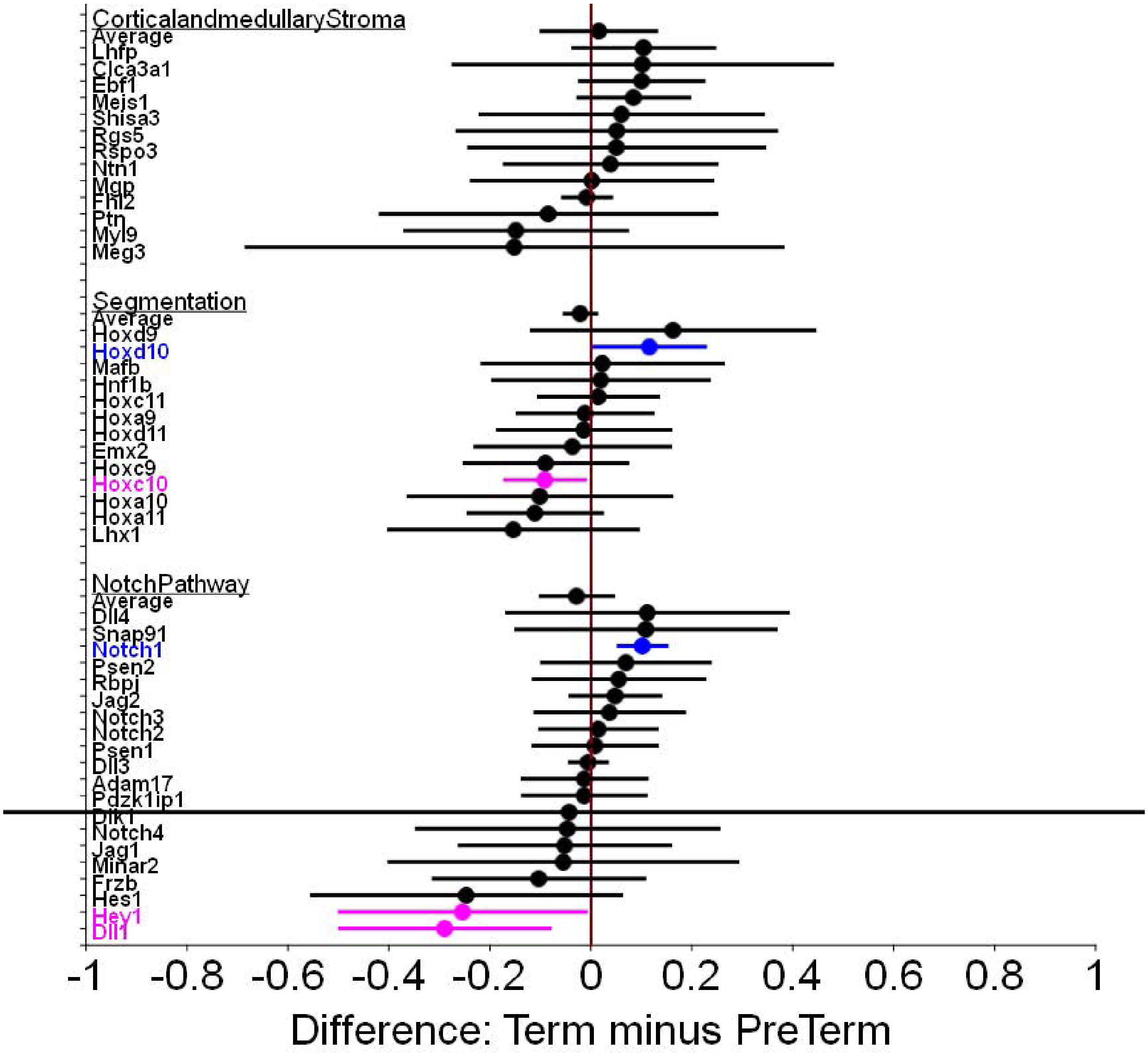

Supplementary Figure S3a. Pathways category part 1 of 3 at 20 dpc.

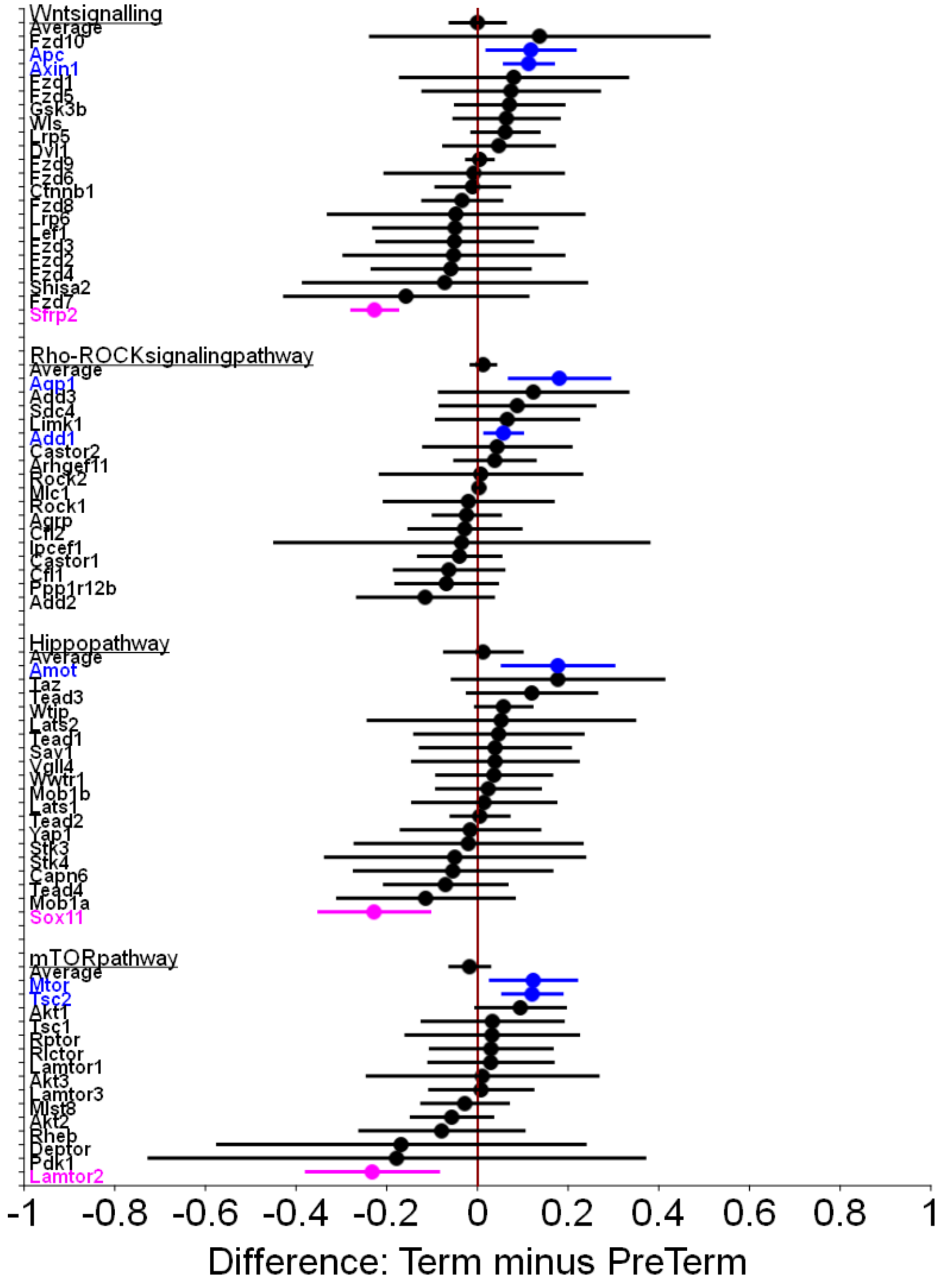

Supplementary Figure S3a. Pathways category part 2 of 3 at 20 dpc.

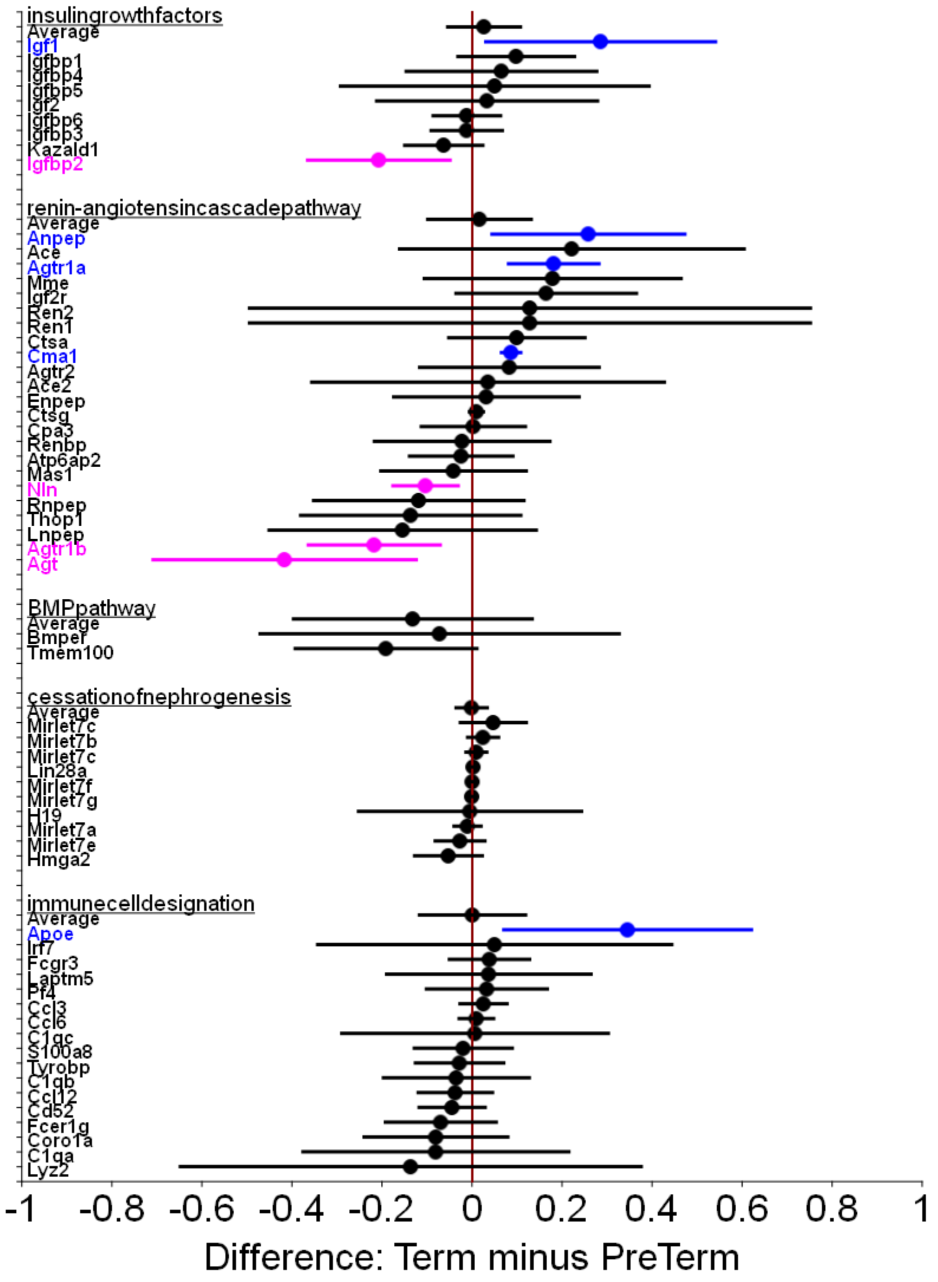

Supplementary Figure S3a. Pathways category part 3 of 3 at 20 dpc.
