# Supplementary material for: Premature differentiation of nephron progenitor cell and dysregulation of gene pathways critical to kidney development in a model of preterm birth
Source: Sci Rep. 2021 Nov 4;11:21667. doi: 10.1038/s41598-021-00489-y (PMC8569166; doi:10.1038/s41598-021-00489-y)
Supplement: Supplementary file 6 — Supplementary Figure S4. [file 41598_2021_489_MOESM6_ESM.docx]

**Supplementary Data: Figure S4**

**Premature differentiation of nephron progenitors and dysregulation of gene pathways critical to kidney development in a model of preterm birth**

Aleksandra Cwiek^1^, Masako Suzuki^3^, Kim deRonde^1^, Mark Conaway^4 5^, Kevin M. Bennett^6^, Samir El Dahr^7^, Kimberly Reidy^2#^, Jennifer R Charlton^1#^*


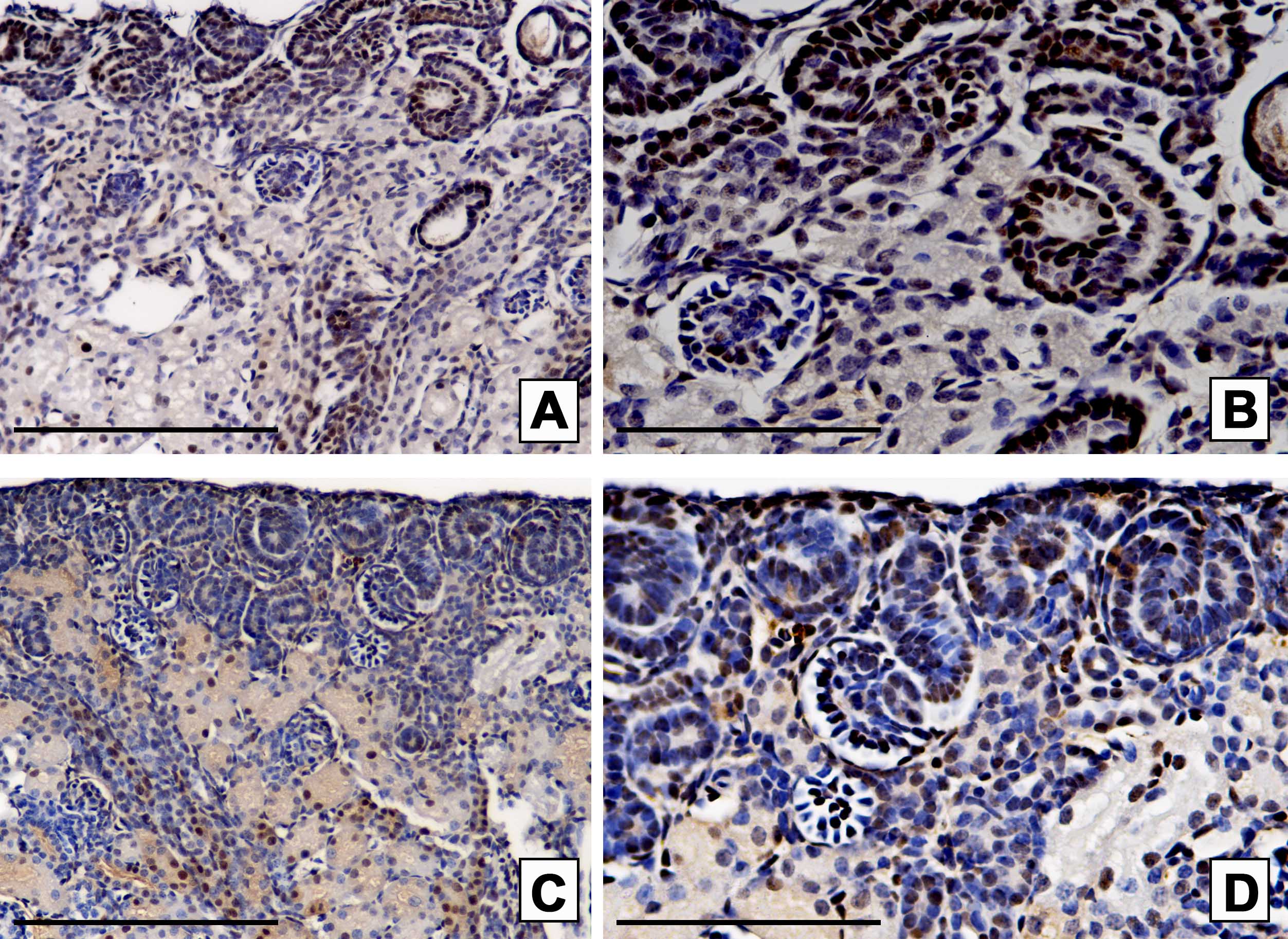


**Supplementary Figure S4. Proliferating cell nuclear antigen staining in the kidney of preterm and term groups at 23 days post conception.** Proliferation in the nephrogenic zone is ongoing in both the preterm and term groups at 23 dpc. In the preterm group, panel A and B, the nephrogenic zone contains developing nephrons with PCNA positive cells, but proliferating cap mesenchyme cells are absent and the zone is narrower than in the term group shown in panels C and D. The nephrogenic zone also appears more disorganized in the preterm group (panela A/B). Scale bar=100 microns.
