# Supplementary material for: Premature differentiation of nephron progenitor cell and dysregulation of gene pathways critical to kidney development in a model of preterm birth
Source: Sci Rep. 2021 Nov 4;11:21667. doi: 10.1038/s41598-021-00489-y (PMC8569166; doi:10.1038/s41598-021-00489-y)
Supplement: Supplementary file 7 — Supplementary Figure S5. [file 41598_2021_489_MOESM7_ESM.docx]

**Supplementary Data: Figure S5**

**Premature differentiation of nephron progenitors and dysregulation of gene pathways critical to kidney development in a model of preterm birth**

Aleksandra Cwiek^1^, Masako Suzuki^3^, Kim deRonde^1^, Mark Conaway^4 5^, Kevin M. Bennett^6^, Samir El Dahr^7^, Kimberly Reidy^2#^, Jennifer R Charlton^1#^*


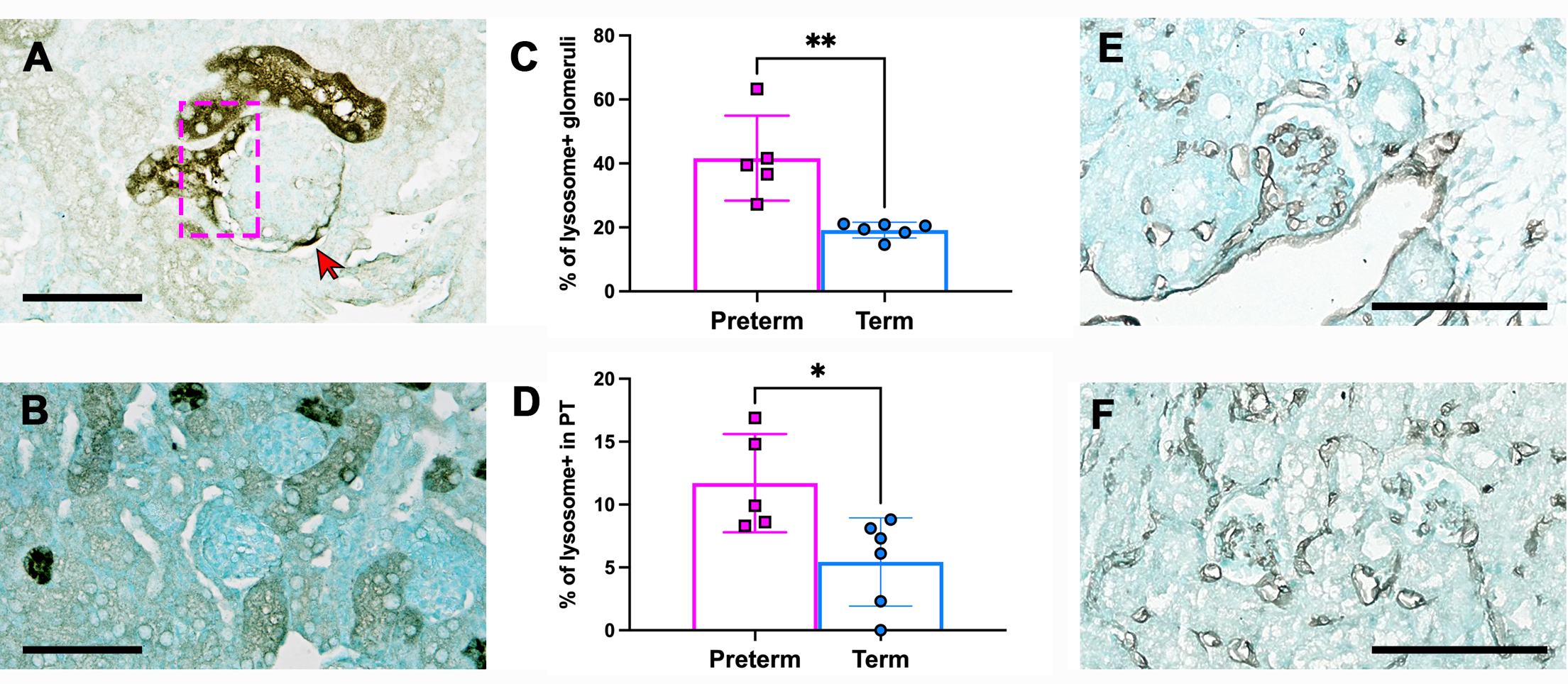


**Supplementary Figure S5.** **Immunohistochemistry confirmation of RNA-Seq data: lysozyme is more common in the glomeruli of the preterm group at 27 dpc.** A representative image of lysozyme detected in the glomerulus of a preterm mouse (red arrow) (A). Lysozyme was more commonly present with the glomeruli of the preterm group (A) as compared to the term (B) group. The majority of glomeruli in the term group did not have lysozyme present (B). The percentage of glomeruli with lysozyme-positive cells is higher in the preterm than term groups (C). Lysozyme was also more commonly detected in the glomerulotubular junction of the preterm as compared to the term group (D). PECAM-1 staining was used to confirm the angiogenesis pathway. The preterm group (E) appeared to have less expression of PECAM-1 in the fully formed glomeruli as compared to the term group (F). Scale bar=100 microns.
