# Supplementary material for: Premature differentiation of nephron progenitor cell and dysregulation of gene pathways critical to kidney development in a model of preterm birth
Source: Sci Rep. 2021 Nov 4;11:21667. doi: 10.1038/s41598-021-00489-y (PMC8569166; doi:10.1038/s41598-021-00489-y)
Supplement: Supplementary file 8 — Supplementary Figure S6. [file 41598_2021_489_MOESM8_ESM.docx]

**Supplementary Data: Figure S6**

**Premature differentiation of nephron progenitors and dysregulation of gene pathways critical to kidney development in a model of preterm birth**

Aleksandra Cwiek^1^, Masako Suzuki^3^, Kim deRonde^1^, Mark Conaway^4 5^, Kevin M. Bennett^6^, Samir El Dahr^7^, Kimberly Reidy^2#^, Jennifer R Charlton^1#^*

**
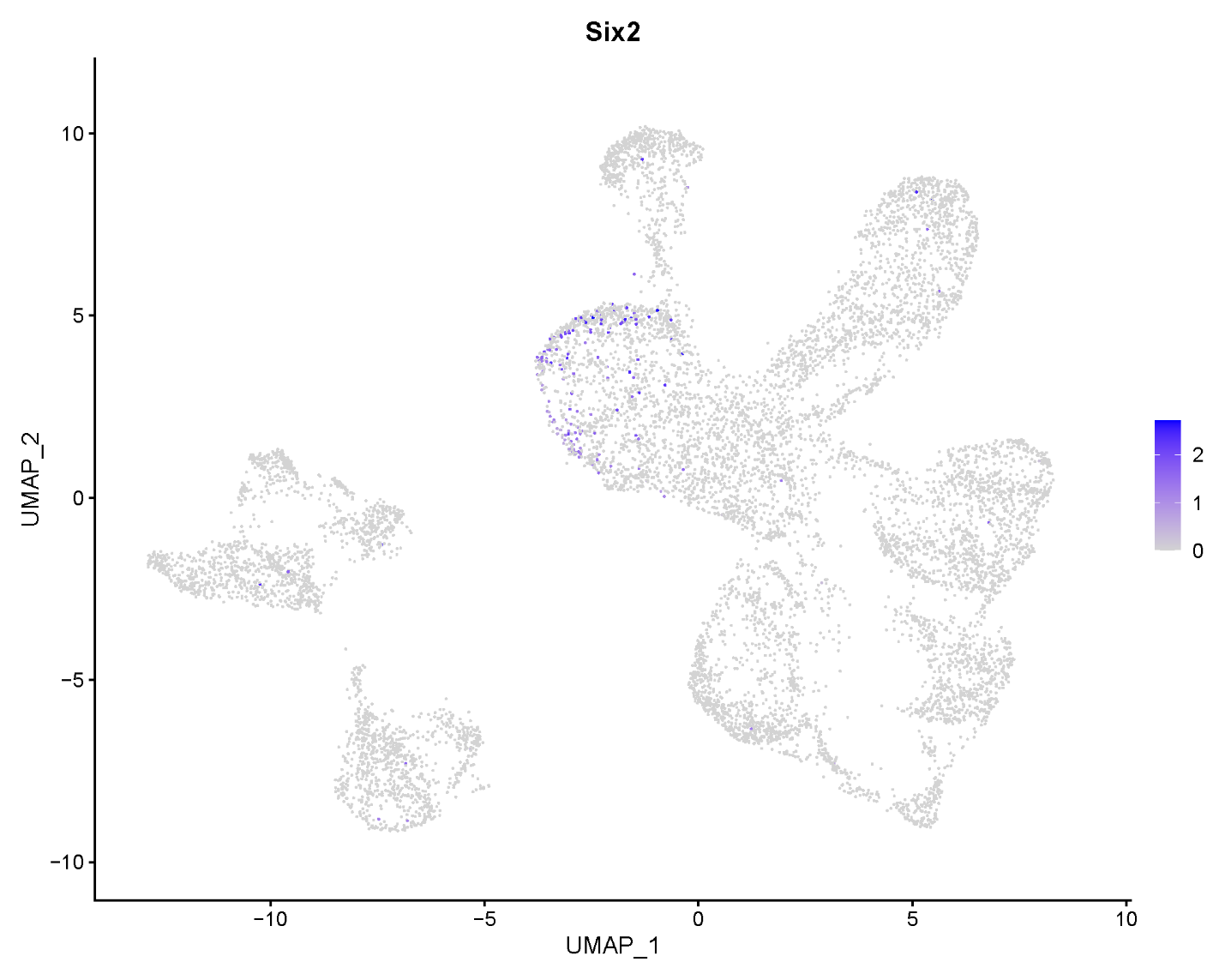
**

**Supplementary Figure S6.** A cell subtype proportion analysis on RNA-seq data – *Six2* in nephron progenitor 1 cluster (NP1).
