# Supplementary material for: Premature differentiation of nephron progenitor cell and dysregulation of gene pathways critical to kidney development in a model of preterm birth
Source: Sci Rep. 2021 Nov 4;11:21667. doi: 10.1038/s41598-021-00489-y (PMC8569166; doi:10.1038/s41598-021-00489-y)
Supplement: Supplementary file 9 — Supplementary Figure S7. [file 41598_2021_489_MOESM9_ESM.docx]

**Supplementary Data: Figure S7**

**Premature differentiation of nephron progenitors and dysregulation of gene pathways critical to kidney development in a model of preterm birth**

Aleksandra Cwiek^1^, Masako Suzuki^3^, Kim deRonde^1^, Mark Conaway^4 5^, Kevin M. Bennett^6^, Samir El Dahr^7^, Kimberly Reidy^2#^, Jennifer R Charlton^1#^*


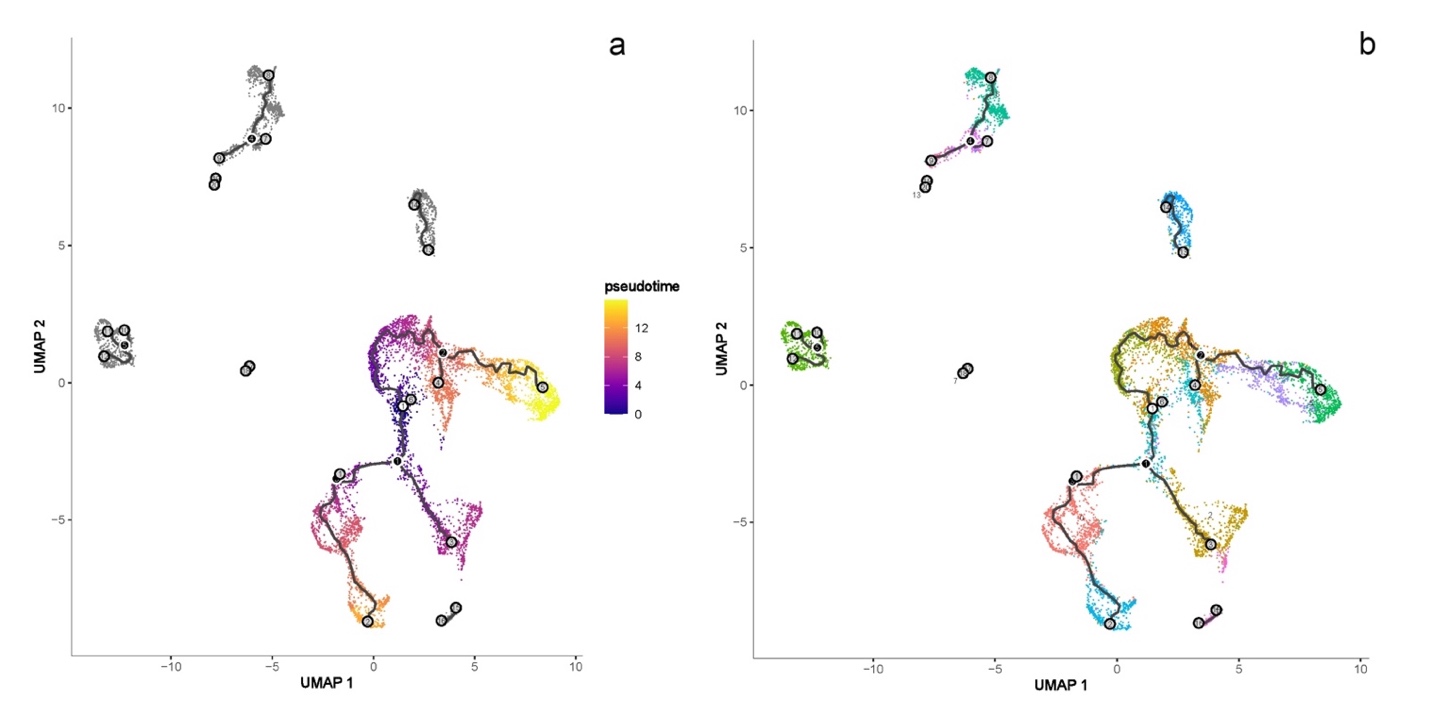


**Supplementary Fig. S7. Pseudotime analysis.** Using data from preterm and term mouse kidneys a 20 dpc, cell expression profiles (marked with single points) were generated in a two-dimensional independent component space. The edges of the minimum spanning tree (MST) created by the Monocle algorithm are indicated by the connected dots. Solid grey line shows the main diameter path of the MST and provides the backbone of Monocle's algorithm pseudotime ordering of the cells. (a) Pseudotime analysis result overlapped with the results from clustering of each cell type (b).
